# Supplementary material for: Reasons for intending to accept or decline kidney cancer screening: thematic analysis of free text from an online survey
Source: BMJ Open. 2021 May 18;11(5):e044961. doi: 10.1136/bmjopen-2020-044961 (PMC8137225; doi:10.1136/bmjopen-2020-044961)
Supplement: Supplementary data [file bmjopen-2020-044961supp001.pdf]

Exploring attitudes towards kidney cancer screening survey

Question	Answer	Validation of question/reference
[Note that this content was delivered online so the formatting looked slightly different to in this document. The participants also did not see the headings in grey boxes or the references, but these are included to illustrate where the questions have come from.]		
Thank you for agreeing to complete this questionnaire. Please answer every question. If you are uncertain about how to answer a question, then please select the closest option.		
Demographic information<br>*In this first section we would like to ask you a few questions about yourself. These questions allow us to make sure we are including people from a range of different backgrounds and see if different groups of people have different views. We will not be able to identify you from your answers.*		
How old are you?	• 45-49<br>• 50-54<br>• 55-59<br>• 60-64<br>• 65-69<br>• 70-74<br>• 75-79	
What is your ethnic group? Choose one option that best describes your ethnic group or background.	• White<br>• Mixed/Multiple ethnic group<br>• Asian/Asian British<br>• Black/African/Caribbean/Black British<br>• Other, please describe______	

What is your highest education level?	• Finished school at or before the age of fifteen<br>• Completed CSEs, O-levels or equivalent<br>• Completed A Levels or equivalent<br>• Completed further education but not a degree<br>• Completed a Bachelor's degree / Masters' degree / PHD<br>• Other (please specify) _______	Validated from Cancer Awareness Measure (CAM) toolkit, (pre-2014, as 2014 onwards has not been validated)[1]
Are you currently:	• Employed full-time<br>• Employed part-time<br>• Unemployed<br>• Self-employed<br>• Full-time homemaker<br>• Retired<br>• Still studying<br>• Disabled or too ill to work<br>• Prefer not to say	Validated from Cancer Awareness Measure (CAM) toolkit, (pre-2014, as 2014 onwards has not been validated)[1]
Please indicate to which occupational group the Chief Income Earner in your household belongs, or which group fits best. This could be you: the Chief Income Earner is the person in your household with the largest income. If the Chief Income Earner is retired and has a pension please answer for their most recent occupation. If the Chief Income Earner is not in paid employment but has been out of work for less than 6 months, please answer for their most recent occupation.	• Professional or higher technical work - work that requires at least degree-level qualifications (e.g. doctor, accountant, schoolteacher, university lecturer, social worker, systems analyst)<br>• Manager or Senior Administrator (e.g. company director, finance	Validated method of determining social class from National Readership Survey, used by Smits et al.,2018 to give ABC1

	manager, personnel manager, senior sales manager, senior local government officer)<br>• Clerical (e.g. clerk, secretary)<br>• Sales or Services (e.g. commercial traveller, shop assistant, nursery nurse, care assistant, paramedic)<br>• Foreman or Supervisor of Other Workers (e.g building site foreman, supervisor of cleaning workers)<br>• Skilled Manual Work (e.g. plumber, electrician, fitter, train driver, cook, hairdresser)<br>• Semi-Skilled or Unskilled Manual Work (e.g. machine operator, assembler, postman, waitress, cleaner, labourer, driver, bar-worker, call centre worker)<br>• Other (Please specify)<br>• Have never worked	(higher SES) and C2DE (lower SES) [2, 3]<br><br>CAM toolkit survey gives examples of occupations which I think fit into the ABC1C2DE framework.[1]
**Kidney cancer screening questions - Information given about kidney cancer potential screening tests**		
Kidney cancer screening tests<br><br>*The following section is about screening for kidney cancer. You will be shown information about kidney cancer and potential screening tests and then asked your views on different screening options.*<br><br>*Kidney cancer is the 7th most common cancer in the UK. There are around 12,600 new kidney cancer cases in the UK every year and 6000 deaths from kidney cancer. In about half of the cases of kidney cancer, there are no obvious symptoms at first. These cases are often picked up during tests*		

carried out for other reasons. Kidney cancer can often be cured, normally by surgery, if it is caught early. However, if it is not diagnosed until it has spread beyond the kidney, then a cure is much less likely. In the UK at the moment nearly a third of all cases of kidney cancer are diagnosed after the kidney cancer had spread beyond the kidneys.

Currently there is no screening programme for kidney cancer. There is potential for a screening programme to be developed in the future though. This may help detect kidney cancer at an early stage, making it easier to treat and reducing the number of people who die from kidney cancer. As with other screening programmes, it would involve having screening tests. If these tests find anything unusual, further tests (usually a detailed CT scan) are needed to confirm a possible kidney cancer. If they find a lump in the kidney then taking a small sample of that may be recommended to confirm or rule out cancer. Some patients wish to have surgery to remove the lump without confirming it is cancer and some of these lumps will not be cancer. As such some people may have an operation that is not required and others will be worried that they have cancer when they don't. There is also a chance that a cancer could be missed as no screening test is 100% reliable. Some people will therefore be told that they don't have cancer when they do.

Over the next few pages you will see some information about each of the potential screening tests. You will then be asked how much inconvenience, burden or worry you associate with each and how likely it is that you would attend for screening with each if you were invited. Please read through the information about the tests carefully so that you can use that information to help you answer those questions.

Ultrasound scan

- *Ultrasound scanning can be used to scan the kidneys. It is similar to the scan offered to pregnant women*
- *Ultrasound scans use high frequency sound waves to make a picture of organs inside your body.*
- *You would need to go to a clinic or hospital for the scan. At the scan you would be asked to lie down and show your abdomen (tummy) by lifting your top. You would not need to undress. A cool gel would be put on your skin.*
- *The ultrasound sensor would be moved over your skin on your lower back, in order to make a picture of each kidney which will appear on a screen.*
- *Ultrasound scans are not painful and there are no risks from the scans themselves. They do not involve exposure to radiation. Most ultrasound scans last between 15 and 45 minutes*
- *If all adults in the UK were offered ultrasound screening, around 3-4 out of every 1000 people screened would have an abnormal scan and require further investigations. Approximately half of those will have kidney cancer.*

Low-dose computed tomography (CT) scan

- *A low-dose computed tomography (CT) scan uses x-rays to make a detailed 3 dimensional (3D) picture of the kidneys. You would need to go to a clinic or hospital for the scan. At the scan you would lie on the machine couch on your back, and the couch slowly moves through the hole of the scanner. The scanner doesn't surround your whole body at once, so you shouldn't feel claustrophobic.*
- *This type of CT scan uses no dyes, no injections, and requires nothing to swallow by mouth*
- *The actual scan takes 1-2 minutes, and the appointment would normally take around 15 minutes.*
- *CT scans are quick, painless and generally safe.*
- *You would be exposed to X-ray radiation during the scan. Generally, the amount of radiation you're exposed to during each scan is the equivalent 6 months of exposure to natural radiation from the environment. It's thought exposure to radiation during CT scans could slightly increase your chances of developing cancer many years later, although this risk is thought to be very small (less than 1 in 2,000).*
- *If all people in the UK were offered screening with CT, around 3-4 out of every 1000 people screened would have an abnormal scan and require further investigations. Approximately half of those will have kidney cancer.*

Urine sample

- *In the future we hope to be able to test for biomarkers in the urine to identify people who are likely to have kidney cancer.*
- *This approach doesn’t diagnose you with kidney cancer, but it is a simple way to find out if you need further tests.*
- *This would require you to provide a urine sample in a pot and either send it off or give to your GP. Your urine sample will then be tested to see if the biomarkers are present.*
- *There are no complications from the test itself but if a test is positive for the kidney cancer biomarkers you may have to have further tests.*
- *If all people in the UK were offered screening with a urine sample, estimates are that around 2-3 out of every 1000 people screened would have an abnormal test and require further investigations. Most of those will have kidney cancer.*

Blood test

- *In the future we hope to be able to test for biomarkers in the blood to identify people who are likely to have kidney cancer.*
- *This approach doesn't diagnose you with kidney cancer, but it is a simple way to find out if you need further tests.*
- *This would require you to have a blood test at your GP's surgery. The blood sample will tested at a laboratory to see if the biomarkers are present.*
- *A blood test can be slightly painful and leave a bruise.*
- *If the test is positive for the kidney cancer biomarkers you may have to have further tests.*
- *If all people in the UK were offered screening with a blood test, around 2-3 out of every 1000 people screened would have an abnormal test and require further investigations. Most of those will have kidney cancer.*

If there was an **ultrasound based screening programme** and you were invited to have an **ultrasound scan** as part of that screening programme for kidney cancer, how likely do you think it is that you would choose to take part in the screening?	• Very likely<br>• Likely<br>• Unlikely<br>• Very unlikely	
Please describe in a few words why?	• Free text:_____	
If there was a **low-dose CT based screening programme** and you were invited to have a **low-dose CT scan** as part of a screening programme for kidney cancer, how likely do you think it is that you would choose to take part in the screening?	• Very likely<br>• Likely<br>• Unlikely<br>• Very unlikely	From Bowel cancer screening preferences survey (ongoing).
Please describe in a few words why?	• Free text:_____	
If there was a **urine sample based screening programme** and you were invited to give a **urine sample** as part of a screening programme for kidney cancer,	• Very likely<br>• Likely<br>• Unlikely	From Bowel cancer screening

how likely do you think it is that you would choose to take part in the screening?	• Very unlikely	preferences survey (ongoing).
Please describe in a few words why?	• Free text:_____	
If there was a **blood sample based screening programme** and you were invited to give a **blood sample** as part of a screening programme for kidney cancer, how likely do you think it is that you would choose to take part in the screening?	• Very likely<br>• Likely<br>• Unlikely<br>• Very unlikely	From Bowel cancer screening preferences survey (ongoing).
Please describe in a few words why?	• Free text:_____	
Thank you for completing the survey. Your time and responses are very much appreciated.		

References

1. Cancer Awareness Measure toolkit version 2.1 Cancer Research UK Cancer Awareness Measure (CAM) Toolkit (version 2.1). 2007. https://www.cancerresearchuk.org/sites/default/files/health_professional_cancer_awareness_measure_toolkit_version_2.1_09.02.11.pdf.

2. Smits SE, McCutchan GM, Hanson JA, Brain KE. Attitudes towards lung cancer screening in a population sample. Heal Expect. 2018; July:1–9.

3. Social Grade | National Readership Survey. http://www.nrs.co.uk/nrs-print/lifestyle-and-classification-data/social-grade/. Accessed 30 May 2019.
