# Supplementary material for: Reasons for intending to accept or decline kidney cancer screening: thematic analysis of free text from an online survey
Source: BMJ Open. 2021 May 18;11(5):e044961. doi: 10.1136/bmjopen-2020-044961 (PMC8137225; doi:10.1136/bmjopen-2020-044961)
Supplement: Author's manuscript [file bmjopen-2020-044961.draft_revisions.pdf]

BMJ Open is committed to open peer review. As part of this commitment we make the peer review history of every article we publish publicly available.

When an article is published we post the peer reviewers' comments and the authors' responses online. We also post the versions of the paper that were used during peer review. These are the versions that the peer review comments apply to.

The versions of the paper that follow are the versions that were submitted during the peer review process. They are not the versions of record or the final published versions. They should not be cited or distributed as the published version of this manuscript.

BMJ Open is an open access journal and the full, final, typeset and author-corrected version of record of the manuscript is available on our site with no access controls, subscription charges or pay-per-view fees (http://bmjopen.bmj.com).

If you have any questions on BMJ Open's open peer review process please email info.bmjopen@bmj.com

BMJ Open

Reasons for intending to accept or decline kidney cancer screening: thematic analysis of free text from an online survey

Journal:	*BMJ Open*
Manuscript ID	bmjopen-2020-044961
Article Type:	Original research
Date Submitted by the Author:	21-Sep-2020

Complete List of Authors:	Freer-Smith, Charlotte; University of Cambridge School of Clinical Medicine<br>Harvey-Kelly, Laragh ; University of Cambridge School of Clinical Medicine<br>Mills, Katie; University of Cambridge, Department of Public Health and Primary Care<br>Harrison, Hannah; University of Cambridge, The Primary Care Unit, Department of Public Health and Primary Care<br>Rossi, Sabrina; University of Cambridge, Department of Surgery; Cambridge University Hospitals NHS Foundation Trust, Department of Urology<br>Griffin, Simon; University of Cambridge, Department of Public Health and Primary Care<br>Stewart, Grant; University of Cambridge, ;<br>Usher-Smith, Juliet; University of Cambridge, Department of Public Health and Primary Care

Keywords:	Kidney tumours < ONCOLOGY, PREVENTIVE MEDICINE, PUBLIC HEALTH, QUALITATIVE RESEARCH

SCHOLARONE™
Manuscripts

I, the Submitting Author has the right to grant and does grant on behalf of all authors of the Work (as defined in the below author licence), an exclusive licence and/or a non-exclusive licence for contributions from authors who are: i) UK Crown employees; ii) where BMJ has agreed a CC-BY licence shall apply, and/or iii) in accordance with the terms applicable for US Federal Government officers or employees acting as part of their official duties; on a worldwide, perpetual, irrevocable, royalty-free basis to BMJ Publishing Group Ltd ("BMJ") its licensees and where the relevant Journal is co-owned by BMJ to the co-owners of the Journal, to publish the Work in this journal and any other BMJ products and to exploit all rights, as set out in our licence.

The Submitting Author accepts and understands that any supply made under these terms is made by BMJ to the Submitting Author unless you are acting as an employee on behalf of your employer or a postgraduate student of an affiliated institution which is paying any applicable article publishing charge ("APC") for Open Access articles. Where the Submitting Author wishes to make the Work available on an Open Access basis (and intends to pay the relevant APC), the terms of reuse of such Open Access shall be governed by a Creative Commons licence – details of these licences and which Creative Commons licence will apply to this Work are set out in our licence referred to above.

Other than as permitted in any relevant BMJ Author's Self Archiving Policies, I confirm this Work has not been accepted for publication elsewhere, is not being considered for publication elsewhere and does not duplicate material already published. I confirm all authors consent to publication of this Work and authorise the granting of this licence.

For peer review only

Reasons for intending to accept or decline kidney cancer screening: thematic analysis of free text from an online survey

Charlotte Freer-Smith[a], Laragh L.W. Harvey-Kelly[a], Katie Mills[b], Hannah Harrison[b], Sabrina H. Rossi[c], Simon J. Griffin [b], Grant D. Stewart[d], Juliet A. Usher-Smith[b]

[a] University of Cambridge School of Clinical Medicine, Addenbrooke's Hospital, Hills Road, Cambridge, CB2 0SP, UK

[b] The Primary Care Unit, Department of Public Health and Primary Care, University of Cambridge, Cambridge, CB2 0SR, UK

[c] Department of Oncology, University of Cambridge, Addenbrooke's Hospital, Cambridge Biomedical Campus, Hills Road, Cambridge CB2 0QQ, UK

[d] Department of Surgery, University of Cambridge, Addenbrooke's Hospital, Cambridge Biomedical Campus, Hills Road, Cambridge CB2 0QQ, UK

Corresponding author:

Dr Juliet Usher-Smith

The Primary Care Unit,

Department of Public Health and Primary Care,

University of Cambridge, Cambridge, CB2 0SR, UK

Tel: 01223 748693

Email: jau20@medschl.cam.ac.uk

Word count: 3938

For peer review only

For peer review only - http://bmjopen.bmj.com/site/about/guidelines.xhtml

ABSTRACT

Objectives: Kidney cancer has been identified as a disease for which screening might provide significant benefit for patients. The aim of this study was to understand in detail the facilitators and barriers towards up-take of a future kidney cancer screening programme, and to compare these across four proposed screening modalities.

Design: An online survey including free text responses.

Setting: UK

Participants: 668 adults

Primary and secondary outcome measures: The survey assessed participants' self-reported intention to take-up kidney cancer screening with four different test methods (urine test, blood test, ultrasound scan, low-dose CT). We conducted thematic analysis of 2559 free text comments made within the survey using an inductive approach.

Results: We identified five overarching themes that influenced screening intention: 'personal health beliefs', 'practicalities', 'opinions of the test', 'attitudes towards screening' and 'cancer apprehension'. Overall participants considered the tests presented as simple to complete and the benefits of early detection to outweigh any drawbacks to screening. Dominant facilitators and barriers varied with patterns of intention to take up screening across the four tests. Most intended to take up screening by all four tests, and for these participants, screening was seen as a positive health behaviour. A significant minority were driven by practicalities and the risks of the tests offered. A smaller proportion intended to reject all forms of screening offered, often due to fear or worry about results and unnecessary medical intervention or a general negative view of screening.

For peer review only - http://bmjopen.bmj.com/site/about/guidelines.xhtml

Conclusions: Most individuals would accept kidney cancer screening by any of the four test options presented because of strong positive attitudes towards screening in general and the perceived simplicity of the tests. Providing information about the rationale for screening in general and the potential benefits of early detection will be important to optimise uptake amongst uncertain individuals.

Key words: Kidney cancer; Cancer; Screening; Survey; Attitudes; Public opinions

For peer review only

Strengths and Limitations of this study

- The free text nature of the questions allowed participants to provide their own reasoning without being prompted or constrained by a list of predefined options
- The large sample size meant that we were able to explore reasons for both intending to take up screening and for not taking up screening
- Our use of an online recruitment platform means the views of those who completed our survey may not be representative of the general population
- In the absence of an existing screening programme we were also only able to assess reasons behind intention and not attendance at screening

For peer review only

For peer review only - http://bmjopen.bmj.com/site/about/guidelines.xhtml

INTRODUCTION

Kidney cancer is the ninth most common cancer worldwide in men and the fourteenth most common cancer in women [1], with over 400,000 new cases being diagnosed in 2018 [2]. The incidence of kidney cancer in the UK is projected to rise by 26% between 2014 and 2035, representing one of the fastest accelerating cancers within that timeframe [3]. Nearly a quarter of individuals diagnosed with kidney cancer in the UK have evidence of metastatic disease at presentation [4]; the five year age-adjusted relative survival is 86% for those diagnosed with the earliest stage of the disease, but drops to 12% for metastatic disease. These epidemiologic data have led to increasing interest in screening for kidney cancer, with the development of a suitable screening programme being identified as a key research priority [5].

For a screening programme to be successful, it must be acceptable to the general public and uptake must be high. Previous research in the context of other screening programmes has identified a number of factors influencing screening uptake. These include practical barriers, for example, difficulty making an appointment or lack of time to do so [67] as well as psycho-social barriers such as fear or embarrassment [7–9]. Identified facilitators of screening attendance encompass both societal factors such as public education, physician recommendation and social networks [10,11], and personal factors such as positive attitudes towards screening [12], the desire for peace of mind [9,13], and subjective assessments of individual health status and cancer risk [11,14]. However, many of these existing studies have identified public opinion through qualitative methods, with small sample sizes, or via surveys using pre-defined lists of possible responses. Furthermore, these studies have focused on cancers for which there are existing national screening programmes (breast,

For peer review only - http://bmjopen.bmj.com/site/about/guidelines.xhtml

cervical and colorectal cancer). Compared to these cancers, kidney cancer has a lower prevalence [3], public awareness of the condition is lower [15], and the range of potential screening modalities is greater. Understanding the specific reasons why people intend to take up or decline kidney cancer screening with different screening tests is therefore important when developing future kidney cancer screening programmes

In an online survey we have shown that a future kidney cancer screening programme would be positively received and intention to attend high, whilst the choice of screening test is likely to have an important influence on uptake (manuscript in submission). The aim of the current study was to use free text responses to questions in the online survey to describe the reasons for intending to accept or decline kidney cancer screening with four different putative screening test options (urine dipstick analysis, blood based biomarkers, low dose computed tomography (CT) and ultrasound). The free text responses are used to understand in detail the reasons for those findings, identify the specific barriers and facilitators for kidney cancer screening, and to compare these drivers across four proposed screening modalities.

METHODS

Study design

This study reports analysis of free text answers from an online population-based survey of 668 members of the public in the UK aged 45-77 years.

Participants and recruitment

For peer review only

Participants were recruited to complete the survey through an online participant recruitment platform for researchers (Prolific, www.prolific.ac). Individuals registered with Prolific were eligible to take part if they were between 45-79 years old and had a Prolific approval rating ≥95% (rating reflects the percentage of studies that participants have completed that have been approved by researchers). The survey was added to the Prolific platform on the 20th of August 2019. At the time of recruitment, 7,767 met the eligibility criteria for the study. Based on demographic data available on the Prolific website, approximately 80% of these were White/Caucasian, 34% male, 53% lived in the UK, 15% were current smokers, 43% had a university level education and 51% considered themselves to be in the top five deciles of socioeconomic status. The survey was live for eight hours. During this time 1,190 participants viewed the participant information leaflet and consent form. A total of 1,021 participants completed the survey. The analysis reported here is based on the free-text comments from the 668 UK based participants. We chose to limit to the UK participants for this analysis to remove any differences in attitudes that may result from different healthcare or insurance systems.

Survey

A copy of the relevant sections of the survey can be found in Supplementary file 1. In brief, after providing basic demographic data, participants were presented with an overview of kidney cancer, and then the details of four potential screening methods (urine testing, blood biomarkers, low-dose CT and ultrasound). This information included the number of people likely to test positive with each test method, and of those, how many people would actually have cancer. These summaries were developed in collaboration with patient and public

For peer review only - http://bmjopen.bmj.com/site/about/guidelines.xhtml

representatives, based on the best available evidence, and the information was reviewed by two urologists within the research team (GDS, SHR). Following presentation of the details of the screening methods, participants were asked how likely they would be to take part in screening with each method (response options were ‘Very likely’, ‘Likely’, ‘Unlikely’ and ‘Very unlikely’). Participants were subsequently asked to “*describe in a few words why*?” they had given that response for each screening modality. This analysis focuses on the free-text responses to those four open-ended questions.

Qualitative Analysis

We conducted thematic analysis of all free text comments using an inductive approach, with initial coding of responses being driven by the content of the comments themselves. Responses were analysed in two groups based on whether participants responded positively (‘likely’ or ‘very likely’ to take part) or negatively (‘unlikely’ or ‘very unlikely’ to take part). From this point, these groups will be referred to as ‘likely’ or ‘unlikely’ respectively. During the analysis process, comments that were inconsistent with the participant’s previous answer were removed (e.g. if they answered ‘very unlikely’ to take part but provided a free text answer that indicated they wanted screening). Incomplete data (where participants did not provide any free text) were also removed.

Two researchers (KM, a female researcher with qualitative expertise and CFS a female medical student with a background in qualitative research) familiarised themselves with a random sample of 400 comments, representing 15% of the total data and equally split between those likely or unlikely to take part, and developed initial codes independently. They then discussed and reviewed the coding decisions and developed two consolidated

For peer review only - http://bmjopen.bmj.com/site/about/guidelines.xhtml

lists: one for the 'likely' to take part comments, and one for the 'unlikely' to take part comments (Figure 1). The remaining free text comments were coded into these agreed lists within an excel file by one researcher (CFS). If a comment contained multiple meanings, the comment was assigned to as many codes as appropriate to cover the content. At this stage, 5% of the total sample, selected at random, was coded by two researchers (CFS and LHK) and any discrepancies discussed. Agreement between the two was 94%. The full list of codes was then discussed at a meeting with the entire research team. Within this meeting, the group mapped the individual codes into overarching themes.

To compare the relative importance of individual codes and themes, the number of times a code was mentioned for each of the four screening tests was counted. We used this data first to explore differences in responses between screening modalities, descriptively highlighting the differences in code frequencies. We then divided participants three subgroups: those likely to take up screening by all four test methods, those unlikely to take up screening by any of the methods, and those whose intention varied between the tests. Differences in the patterns of responses between these groups were explored. Finally, we investigated the patterns of responses with sex, age, level of education, and social group.

Ethics approval and consent to participate

Ethical approval was obtained from the Psychology Research Ethics committee of the University of Cambridge (Ref 2019.055). All participants gave informed written online consent before they began the survey.

For peer review only

Patient and Public involvement

Two members of the public contributed, through face-to-face discussions and email, to the development of the survey and participant information sheet. They also provided comments on the final manuscript prior to submission.

RESULTS

The sociodemographic profile of the sample is shown in Table 1. 373 (54%) were female, with a mean age of 54.7 ± 7 years and a good distribution across age groups. Most (73%) participants were in social group ABC1 (those with managerial, administrative, or professional job roles) and 288 (43.1%) had university level education.

Intention to take up kidney cancer screening

Overall, the majority of participants (518, 78%) were likely to take up screening by all four screening test modalities. Of the remaining participants, 119 (18%) would be likely to take up some screening tests but not all, with 55/119 (46%) likely to attend for an ultrasound, urine or blood testing, but not for a low-dose CT scan. 31 (5%) indicated that they were unlikely to take up screening by any of the test methods. Since intention to take-up screening was high, we collected a greater number of free-text comments relating to reasons why someone was likely to take part in screening. Once inconsistent and blank data had been removed (51 and 62 participants respectively) there were 2304 comments from those 'likely' to take up screening and 255 comments from those 'unlikely' to take up a screening.

For peer review only - http://bmjopen.bmj.com/site/about/guidelines.xhtml

Themes identified

From the free text comments, we identified 49 individual codes (27 facilitators and 22 barriers from those likely and unlikely to take up screening respectively). On average, each comment was coded under 1.5 codes: 1,448 (56.6%) of comments were relevant to one code, 907 (35.4%) were relevant to two codes, with the remaining 204 (8%) relevant to 3 or more. Together the codes mapped onto five overarching themes: Personal health beliefs; cancer apprehension; attitudes towards screening; opinions of the test; and practicalities (additionally, we included an 'other' category containing five codes that did not map elsewhere). All five themes included codes from both facilitators and barriers: the full list of these codes, mapped onto each relevant theme, is presented in Figure 1. Illustrative quotes from each theme are presented in Table 2.

Facilitators and barriers identified across screening methods

Table 3 shows the frequency with which each of the facilitators and barriers were reported, overall and across the four screening modalities. 648 participants provided comments relating to blood tests. 589 (91%) of those were likely to attend. Comments amongst that group focused primarily on statements that blood testing is '*simple to complete*' (160, 25%), the '*benefits of screening outweigh the drawbacks*' (84, 13%) and '*early detection is a benefit*' (73, 11%). Of those unlikely to take up blood testing (59, 9%), the only consistently mentioned barrier was a '*dislike of the test method'*, mentioned by 3% (21).

Among the 637 participants providing comments on urine testing, 608 (95%) were likely to take up screening. Similar to the responses for blood testing, the most common overall

For peer review only - http://bmjopen.bmj.com/site/about/guidelines.xhtml

For peer review only

reason for taking up screening was that the test is '*simple to complete*' (282, 44%). Frequently cited other comments that related specifically to the characteristics of the test were '*little or no inconvenience*' (79, 12%) and '*quick*' (57, 9%). *'Early detection is a benefit'* was also frequently mentioned by this group (63, 10%) but references to *'benefits of screening outweigh the drawbacks'* were less common, potentially reflecting that fewer drawbacks are associated with urine testing than blood testing. Overall, no single barrier to urine testing was identified, with no code occurring more than 6 times.

631 participants provided comments relating to ultrasound scans and 643 relating to CT. Intention to take up screening was again high with 575 (91%) likely to attend ultrasound and 532 (82%) CT. Notably, among participants likely to accept screening by ultrasound or CT the most mentioned facilitator for both does not relate to the test itself but the belief that *'early detection is a benefit'*, (97 (15%) for CT scan, 107 (17%) for ultrasound). Although less dominant, *'simple to complete'* was still mentioned for both (55 (9%) for CT, 80 (13%) for ultrasound). For ultrasound, there are also multiple codes within the theme 'opinions of the test' that are mentioned more frequently than for other test methods: *'painless'* (57, 9%) '*non-invasive'* (53, 8%) and *'without risks'* (46, 7%). Of these facilitators, only *'painless'* is mentioned with some frequency for CT scans (45, 7%). As seen for blood tests, *'benefits of screening outweigh the drawbacks'* is also a dominant facilitator for CT scans.

The key barrier identified by those unlikely to take up a CT scan is the *'risk of the test'* (53, 8%), some mentioning only concerns regarding the danger of radiation, while others linked this to long term cancer risks. An additional barrier highlighted for both CT and ultrasound, but not blood or urine testing, was travel to a hospital (10 (2%) and 9 (1%) respectively).

For peer review only - http://bmjopen.bmj.com/site/about/guidelines.xhtml

Notably, across all test modalities and across those likely or unlikely to attend screening, there were very few comments that related to the risk of kidney cancer specifically or the accuracy of the tests. Evaluation of the responses by demographics also revealed no clear patterns with age, sex, social group or university education.

Subgroup thematic analysis

Comparison of the comments made by participants who said they were likely to take up screening by all four test methods with those who would accept screening by some of the test methods only is presented in Table 4. For those whose likelihood of taking up screening varied between the different tests, 'practicalities' and 'opinions of the test' remained the most prominent themes. *'Simple to complete'* represented the most frequently mentioned individual code (41%, n=118/290 comments), with most of these from comments in relation to urine or blood testing. The non-invasive nature of ultrasound scanning, and perceived lack of risks associated with this test were also seen in this group.

Conversely, for participants who would accept screening with all four tests, the themes 'attitudes towards screening', 'cancer apprehension' and 'personal health beliefs' are proportionally more important. Whilst *'simple to complete'* is still the most mentioned individual code, others within these additional themes become equally important. The facilitators identified within the 'attitudes towards screening' theme reflect a general attitude that screening is a "correct" form of behaviour: *'sensible'* (10%, n=200/2014 comments), *'for my general health / good'* (6%, n=115/2014), *'early detection is a benefit'* (16%, n=323/2014 comments). The perceived benefits of early detection that were highlighted by participants included improved survival, better treatment, and more

For peer review only - http://bmjopen.bmj.com/site/about/guidelines.xhtml

treatment options. Many participants, however, simply stated that early detection is always beneficial and did not give further explanation. Another common belief described by this group was the idea that screening could provide a sense of *'peace of mind / reassurance'* that they did not have the disease (11%, n=219/2014), highlighting one way in which cancer worry can motivate screening attendance.

Table 5 shows the barriers cited by those who are unlikely to take up screening by any of the tests and those whose attitude towards screening varied with screening modality. For those declining some test methods, but not all, the only dominant theme is 'opinions of the test': most of these comments reference the *'risks of the test'* for CT scan (54%, 45/84), or a *'dislike of the test'* for blood tests (59%, 19/32). Very few other barriers outside of these are mentioned. In contrast, those who decline all screening methods do not mention test specific concerns, instead 'attitudes towards screening', 'personal health beliefs' and 'cancer apprehension' are all more frequently raised across all the test modalities. The most frequently mentioned individual barrier to screening is *'worry / fear about results'* (17%, 18/109). Many also state that they '*do not want any screening'* (15%, 16/109) or they believe that that '*medical care is for diagnosis / symptoms only*' (14%, 15/109), reflecting a disagreement with the principle of screening in general.

DISCUSSION

Key Findings

Through a thematic analysis of over 2000 free text comments from an online survey, we have identified, and ranked the relative importance of, facilitators and barriers influencing

For peer review only - http://bmjopen.bmj.com/site/about/guidelines.xhtml

potential uptake of kidney cancer screening by four possible screening modalities. We found that, overall, across all four tests most participants considered screening simple to complete and that the benefits of early detection outweigh any burdens or harms. Urine testing was viewed as the simplest and least invasive to complete; without the risks or inconvenience associated with other tests. Additionally, we found that the dominant drivers and barriers varied with patterns of intention to take up screening across the four tests. The most frequent pattern, and therefore the largest group of individuals, was those who were likely to take up screening with all four tests. Amongst this group, screening was seen as a positive health behaviour, with many benefits: the tests are seen as simple, they see early detection as an advantage, or seek the peace of mind and clarity that accompanies a negative screening result. The second group was those for whom the specific test modality influences decision making. Within this group a significant minority would attend all tests except for a CT scan, due to concern about the risk associated with a CT. Blood tests also polarised participants: most were comfortable with this method and it was seen as simple by many, but a small minority expressed a specific dislike of blood tests. Having to travel to hospital for a CT or USS also put some people off these modalities. The smallest group was those who reject all forms of screening. For these individuals, the drawbacks of screening in general are highlighted rather than opinions related to specific tests. These drawbacks include fear or worry about results and unnecessary medical intervention and are not balanced by sufficient benefits. Notably, largely absent from the reasons that participants did or did not intend to take up screening among any of these groups were comments related to the accuracy of the tests or the risk of kidney cancer specifically, despite information on both of these being included within the survey.

For peer review only - http://bmjopen.bmj.com/site/about/guidelines.xhtml

Comparison with existing literature

Our finding that the decision to take up screening in this study, for many of the participants, was driven by the perceived value of early detection, with screening embodying a positive health behaviour (or a sensible decision) is consistent with previous studies exploring facilitators of screening in existing cancer screening programmes [10,11,13,16–18] and the view that screening is 'almost always a good idea'[19]. Our study builds on these findings by showing that these general beliefs about screening extend to hypothetical new screening programmes and, for the individuals who hold these views, the nature of the screening test is not a key factor in their decision of whether to take up screening or not. This observation, that screening decisions are often driven by emotions and strongly held contextual beliefs rather than specific cognitive processes, was also seen among those who did not intend to attend screening with any of the tests. While a small number of participants in this group appear to have been consciously weighing up the potential benefits and harms of kidney cancer screening within the context of their own personal health beliefs, our findings suggest many may be driven by a more general aversion to the concept of screening or apprehension about cancer in general or the results. This has been reported in the context of other screening programmes [12,20–24] and highlights the need for information materials to clarify the purpose of screening and advantages of early diagnosis in asymptomatic individuals [21] and to specifically address negative beliefs around the disease and cancer worry. The preference for simple and convenient tests, seen through the responses for urine and blood testing in particular in this study, has also been reported previously in the context of other cancer screening programmes [7,14,25]. Our findings additionally show that the risks associated with exposure to radiation for CT are a key barrier for some individuals.

For peer review only - http://bmjopen.bmj.com/site/about/guidelines.xhtml

Strengths and Limitations

The main strengths of this study are the large sample size and the free text nature of the questions that allowed participants to provide their own reasoning without being prompted by a list of predefined options.

The large sample size meant that we were able to explore reasons for not taking up screening among the minority of participants who would be unlikely to take it up. Uptake rates for UK cancer screening programmes are consistently below 75% [26–28], therefore, capturing the perspectives of those who are less likely to accept screening (and difficult to engage in research) is important. Since we were also only able to assess the reasoning behind the participants' intention to attend screening, and not their actual attendance, this highlights a limitation of our study, as intention may be higher than attendance.

As a result of our use of an online recruitment method, however, the views of those who completed our survey may not be representative of the general population [29]. In particular, the distribution across social groups was different: 73% of our participants reported being in the upper half of the social grades (ABC1), whereas census data from 2011 suggests 53% of the UK population lies within this category. Given that participation in screening programmes is disproportionately lower among lower social grades [18,30], the participants in our study may, therefore, be more enthusiastic about screening than the UK population as a whole. With online data collection there is also the risk that participants did not concentrate and their responses do not accurately reflect their views. We sought to limit this by including an instructional manipulation check early on the questionnaire. Only 51 of the

For peer review only - http://bmjopen.bmj.com/site/about/guidelines.xhtml

2,559 comments (1.9%) were also inconsistent, suggesting that the overwhelming majority of participants were appropriately engaged with the survey.

The free text nature of the questions meant that participants were free to express their thoughts in their own words. This has the advantage of not constraining them by pre-defined lists. However, the question we asked ("*Please describe in a few words why*?") did not instruct participants to list all the reasons behind their choice, meaning responses ranged from one word to several sentences. Our findings, therefore, reflect the dominant views of participants when faced with the option of a given screening test, not all their views. The relative frequency of the different codes and themes reflects their importance and not the necessarily the number of participants for whom that factor may contribute to their decision.

CONCLUSIONS

Our findings show that most individuals think they are likely to attend kidney cancer screening by any of the four test options presented. These individuals are driven both by a general perception of screening as a good thing and the view that the tests are simple, making them likely to participate in any future kidney cancer screening programme largely without consideration of the nature of the test involved or their own risk of kidney cancer. For the significant minority for whom the practicalities and risks of the tests are important, optimising the logistics of the screening programme and ensuring that the written information is understandable, for example around the radiation risk associated with CT, will be important. Providing sufficient information to enable individuals to make an informed choice about screening will also be important for the small proportion of the

For peer review only - http://bmjopen.bmj.com/site/about/guidelines.xhtml

population who do not intend to take up screening with any of the test options. Our findings suggest that that information should not be limited to the specific details of kidney cancer screening but should also include an explanation of the rationale for screening in general and the potential benefits of early detection.

Acknowledgements

We would like to thank our two patient and public representatives Phil Alsop and Philip Dondi for their input and advice throughout this study.

Authors' contributions

JUS, LHK, KM, HH, SHR, GDS and SG were involved in the design of the study. JUS completed data collection. CFS, KM, LHK were involved in initial data analysis, and all authors contributed to the final analysis and interpretation of the data. CFS and JUS wrote the first draft of the manuscript. All authors critically reviewed the manuscript and have approved the final version.

Funding

This work was funded by a research grant from Kidney Cancer UK. JUS and KM were supported by a Cancer Research UK Cancer Prevention Fellowship (C55650/A21464). SHR is supported by The Urology Foundation and a Cancer Research UK Clinical Research Fellowship. GDS is funded by the Cancer Research UK Cambridge Cancer Centre (Major

For peer review only - http://bmjopen.bmj.com/site/about/guidelines.xhtml

For peer review only

Centre Award A25117) and the Renal Cancer Research Fund. HH is supported by a National Institute of Health Research Methods Fellowship (RM-SR-2017-09-009). The University of Cambridge has received salary support in respect of SJG from the NHS in the East of England through the Clinical Academic Reserve.

The views expressed in this publication are those of the author(s) and not necessarily those of the NHS, the National Institute for Health Research or the Department of Health and Social Care

Data sharing

The dataset supporting the conclusions of this article will be made available in the University of Cambridge data repository (https://www.repository.cam.ac.uk/) for at least 10 years from the last access. All the data will be stored in accordance with the Data Protection Act 1998.

Financial disclosure: All authors have completed the Unified Competing Interest form at www.icmje.org/coi_disclosure.pdf (available on request from the corresponding author). GDS has received educational grants from Pfizer, AstraZeneca and Intuitive Surgical; consultancy fees from Pfizer, Merck, EUSA Pharma and CMR Surgical; Travel expenses from Pfizer and Speaker fees from Pfizer. All other authors declare that (1) they have no support from or relationships with companies that might have an interest in the submitted work in the previous 3 years; (2) their spouses, partners, or children have no financial relationships

For peer review only

that may be relevant to the submitted work; and (3) they have no non-financial interests that may be relevant to the submitted work.

The corresponding author has the right to grant on behalf of all authors and does grant on behalf of all authors, an exclusive licence on a worldwide basis to the BMJ Publishing Group Ltd and its Licensees to permit this article (if accepted) to be published in BMJ editions and any other BMJPGL products and sub-licences to exploit all subsidiary rights, as set out in our licence (http://resources.bmj.com/bmj/authors/checklists-forms/licence-for-publication).

All authors had full access to all of the data in the study and can take responsibility for the integrity of the data and the accuracy of the data analysis

The corresponding author affirms that the manuscript is an honest, accurate, and transparent account of the study being reported; that no important aspects of the study have been omitted; and that any discrepancies from the study as planned (and, if relevant, registered) have been explained.

For peer review only

REFERENCES

1 Znaor A, Lortet-Tieulent J, Laversanne M, *et al.* International Variations and Trends in Renal Cell Carcinoma Incidence and Mortality. *Eur Urol* 2015;**67**:519–30. doi:10.1016/j.eururo.2014.10.002

2 Bray F, Ferlay J, Soerjomataram I, *et al.* Global cancer statistics 2018: GLOBOCAN estimates of incidence and mortality worldwide for 36 cancers in 185 countries. *CA Cancer J Clin* 2018;**68**:394–424. doi:10.3322/caac.21492

3 Smittenaar CR, Petersen KA, Stewart K, *et al.* Cancer incidence and mortality projections in the UK until 2035. *Br J Cancer* 2016;**115**:1147–55. doi:10.1038/bjc.2016.304

4 Cancer survival in England - Office for National Statistics. https://www.ons.gov.uk/peoplepopulationandcommunity/healthandsocialcare/conditionsanddiseases/bulletins/cancersurvivalinengland/nationalestimatesforpatientsfollowedupto2017#:~:text=Cancer survival in children (aged,year and 10-year survival. (accessed 10 Jul 2020).

5 Rossi SH, Fielding A, Blick C, *et al.* Setting Research Priorities in Partnership with Patients to Provide Patient-centred Urological Cancer Care. Eur. Urol. 2019;**75**:891–3. doi:10.1016/j.eururo.2019.03.008

6 Waller J, Bartoszek M, Marlow L, *et al.* Barriers to cervical cancer screening attendance in England: a population-based survey. 2009;**16**:199–204.

7 Aubin-Auger I, Mercier A, Lebeau J-P, *et al.* Obstacles to colorectal screening in general practice: a qualitative study of GPs and patients. *Fam Pract* 2011;**28**:670–6.

For peer review only - http://bmjopen.bmj.com/site/about/guidelines.xhtml

doi:10.1093/fampra/cmr020

8 Bukowska-Durawa A, Luszczynska A. Cervical cancer screening and psychosocial barriers perceived by patients. A systematic review. Wspolczesna Onkol. 2014;**18**:153–9. doi:10.5114/wo.2014.43158

9 Ritvo P, Myers RE, Paszat L, *et al.* Gender differences in attitudes impeding colorectal cancer screening. *BMC Public Health* 2013;**13**:1. doi:10.1186/1471-2458-13-500

10 Young B, Bedford L, Kendrick D, *et al.* Factors influencing the decision to attend screening for cancer in the UK: a meta-ethnography of qualitative research. *J Public Health (Bangkok)* 2017;**40**:315–39. doi:10.1093/pubmed/fdx026

11 Honein-abouhaidar GN, Kastner M, Vuong V, *et al.* Systematic Review and Meta-study Synthesis of Qualitative Studies Evaluating Facilitators and Barriers to Participation in Colorectal Cancer Screening. 2016;:907–18. doi:10.1158/1055-9965.EPI-15-0990

12 Smits SE, McCutchan GM, Hanson JA, *et al.* Attitudes towards lung cancer screening in a population sample. *Heal Expect* 2018;:1–9. doi:10.1111/hex.12819

13 Robb KA, Solarin I, Power E, *et al.* Attitudes to colorectal cancer screening among ethnic minority groups in the UK. *BMC Public Health* 2008;**8**. doi:10.1186/1471-2458-8-34

14 Edgar L, Glackin M, Hughes C, *et al.* Factors influencing participation in breast cancer screening. *Br J Nurs* 2013;**22**:1021–6.

15 Public awareness of cancer in Britain: Report for the National Awareness and Early Diagnosis Initiative. 2009 Opinions Survey. https://www.cancerresearchuk.org/sites/default/files/public_awareness_of_cancer_i

For peer review only - http://bmjopen.bmj.com/site/about/guidelines.xhtml

n_britain_dh_report.pdf

16 Dam L Van, Korfage IJ, Kuipers EJ, *et al.* What influences the decision to participate in colorectal cancer screening with faecal occult blood testing and sigmoidoscopy ? *Eur J Cancer* 2013;**49**:2321–30. doi:10.1016/j.ejca.2013.03.007

17 James LJ, Wong G, Craig JC, *et al.* Men's perspectives of prostate cancer screening: A systematic review of qualitative studies. *PLoS One* 2017;**12**:e0188258. doi:10.1371/journal.pone.0188258

18 Pfeffer N. Screening for breast cancer: Candidacy and compliance. *Soc Sci Med* 2004;**58**:151–60. doi:10.1016/S0277-9536(03)00156-4

19 Waller J, Osborne K, Wardle J. Enthusiasm for cancer screening in Great Britain: a general population survey.[Erratum appears in Br J Cancer. 2016 Jun 14;114(12):e14; PMID: 27228290]. *Br J Cancer* 2015;**112**:562–6.

20 Harte E, Maclure C, Martin A, *et al.* Reasons why people do not attend NHS Health Checks: A systematic review and qualitative synthesis. *Br J Gen Pract* 2017;:1–8. doi:https://doi.org/10.3399/bjgp17X693929

21 Palmer CK, Thomas MC, von Wagner C, *et al.* Reasons for non-uptake and subsequent participation in the NHS Bowel Cancer Screening Programme: a qualitative study. *Br J Cancer* 2014;**110**:1705–11. doi:10.1038/bjc.2014.125

22 Johnson TP, O??Rourke DP, Burris JE, *et al.* An Investigation of the Effects of Social Desirability on the Validity of Self-Reports of Cancer Screening Behaviors. *Med Care* 2005;**43**:565–73. doi:10.1097/01.mlr.0000163648.26493.70

23 Allen JD, Bluethmann SM, Sheets M, *et al.* Women's responses to changes in U.S.

For peer review only - http://bmjopen.bmj.com/site/about/guidelines.xhtml

preventive task force's mammography screening guidelines: Results of focus groups with ethnically diverse women. *BMC Public Health* 2013;**13**. doi:10.1186/1471-2458-13-1169

24 Rosenbaum L. Invisible Risks, Emotional Choices — Mammography and Medical Decision Making. *N Engl J Med* 2014;**371**:175–7.

25 Waller J, Bartoszek M, Marlow L, *et al.* Barriers to cervical cancer screening attendance in England: a population-based survey. *J Med Screen* 2009;**16**:199–204. doi:10.1258/jms.2009.009073

26 Health and Social Care Information Centre. Cervical Screening Programme, England – 2015-2016. https://digital.nhs.uk/data-and-information/publications/statistical/cervical-screening-annual/cervical-screening-programme-england-2015-2016 (accessed 10 Jul 2020).

27 Health and Social Care Information Centre. Breast Screening Programme, England – 2016-17. https://digital.nhs.uk/data-and-information/publications/statistical/breast-screening-programme/breast-screening-programme-england---2016-17#:~:text=Women between the ages of,better chance of successful treatment. (accessed 10 Jul 2020).

28 Logan RFA, Patnick J, Nickerson C, *et al.* Outcomes of the Bowel Cancer Screening Programme (BCSP) in England after the first 1 million tests. *Gut* 2012;**61**:1439–46. doi:10.1136/gutjnl-2011-300843

29 Palan S, Schitter C. Prolific.ac—A subject pool for online experiments. *J Behav Exp Financ* 2018;**17**:22–7. doi:10.1016/j.jbef.2017.12.004

For peer review only - http://bmjopen.bmj.com/site/about/guidelines.xhtml

30 McCaffery K, Wardle J, Nadel M, *et al.* Socioeconomic variation in participation in colorectal cancer screening. *J Med Screen* 2002;**9**:104–8. doi:10.1136/jms.9.3.104

FIGURE LEGENDS

Figure 1. Individual codes (facilitators and barriers) mapped onto overarching themes

SUPPLEMENTARY FILES

Supplementary File 1. Exploring attitudes towards kidney cancer screening survey (PDF 907kb)

For peer review only

Table 1. Participant characteristics

	UK (N=668)<br>n (%), mean (SD)
**Age**	
45-49	188 (28)
50-54	195 (29)
55-59	123 (18)
60-64	91 (14)
≥65	71 (11)
Mean (SD)	54.7 (7.0)
**Sex**	
Female	373 (54)
Male	295 (44)
**University education**	
Yes	288 (43)
No	380 (57)
**Ethnicity**	
White	653 (98)
Other	15 (2)
Missing	0
**General health measure**	
Excellent, very good, good	524 (78)
Fair, poor	144 (22)
**Smoking**	
Non-smoker	340 (51)
Ex-smoker	236 (35)
Current smoker	92 (14)
**BMI**	
Mean (SD)	27.4 (5.8)
Range	16.5-50.0
Missing	29 (4)
**Previous diagnosis of cancer?**	
Yes	34 (5)
No	634 (95)
**Family history of kidney cancer?**	
Yes	19 (3)
No	635 (95)
Missing	14 (2)
**Social group**	
ABC1	500 (75)
C2DE	136 (20)
Missing	32 (5)

For peer review only

Table 2. Illustrative quotes of themes

Qualitative theme	Barriers: Illustrative quotes	Facilitators: Illustrative quotes
**Personal health beliefs**	*"I have a healthy lifestyle and at a low risk for kidney cancer." (F, 55-59y, ultrasound)*<br>*"There is no kidney cancer or cancer in my immediate family." (F, 45-49y, blood test)*	*"As my parent had kidney cancer, I would very much put myself forward to screening." (F, 60-64y, ultrasound)*<br>*"Because life is precious, I want to live as long as possible." (M, 55-59y, CT)*
**Practicalities**	*"Because my mobility problems make it very difficult for me to travel anywhere." (F, 55-59y, ultrasound)*<br>*"If it involved a specific visit, it's unlikely I would want to go." (M, 55-59y, urine test)*	*"It's no inconvenience at all to have any of the screening methods discussed" (M, 50-54y, urine test)*<br>*"I have had ultrasound scans before, its quick and easy" (F, 45-49y, ultrasound scan)*
**Opinions of the test**	*"I would not wish to receive a dose of radiation to test for the extremely small chance of kidney cancer." (M, 65-69y, CT)*<br>*"Really don't like needles, I'd opt for something else if it were available." (M, 45-49y, blood tests)*	*"A simple procedure and blood tests don't really alarm me at all" (M, 70-74y, blood test)*<br>*"Easy to do when attending the doctors for something else and not painful in anyway" (F, 50-54y, urine test)*
**Attitudes towards screening**	*"Generally, I do not like medical appointments unless I need treatment." (M, 55-59y, blood tests)*<br>*"There are a lot of cancers, you can't screen for all of them. If I had symptoms, I would see a GP." (F, 50-54y, ultrasound)*	*"Benefit of early detection outweighs minor inconvenience of screening" (M, 55-59y, blood test)*<br>*"Would be foolish not to. If the disease has no symptoms and can be identified through screening, has the potential to save lives." (F, 55-59y, CT)*
**Cancer apprehension**	*"I would be too afraid of the results. Short of a transplant, what could be done anyway?" (F, 55-59y, urine test)*<br>*"The stress of going to the hospital waiting for results is too great." (F, 60-64y, CT)*	*"Why waste a chance to 'set nerves at rest'?" (M, 55-59y, blood test)*<br>*"High value of early detection or peace of mind" (M, 50-54y, blood test)*

For peer review only - http://bmjopen.bmj.com/site/about/guidelines.xhtml

Table 3. Facilitators and barriers of screening uptake across modalities, frequency of mention of codes and themes. The colour gradients shown indicate where each value falls within the range of data shown calculated within either the facilitator, or the barrier dataset. USS – ultrasound; HCP – healthcare professional; tx - treatment

**Themes**	**Individual Codes**	Blood	Urine	CT	USS	Total	Theme total
		n=589	*n*=608	*n*=532	*n*=575	*n*=2304	
Personal Health beliefs	Life Extending / Improved survival	34	34	54	48	**204**	**224**
	Family history of cancer	2	1	2	3	**9**	
	Risk of kidney cancer	0	1	2	7	**11**	
Practicalities	Simple to complete	160	282	55	80	**859**	**1468**
	Little or no inconvenience	38	79	26	32	**254**	
	Test can be conducted at GP	22	32	0	4	**90**	
	Quick	42	57	32	43	**231**	
	Cost effective	8	10	4	2	**34**	
Opinions of the test	Painless	18	26	45	57	**172**	**803**
	Test non-invasive	6	23	27	53	**132**	
	Test is without risks	21	23	23	46	**136**	
	Comfortable having test	52	17	17	23	**126**	
	Have had test before	34	15	20	39	**123**	
	Effective test	25	24	14	13	**100**	
	Low radiation	0	1	10	2	**14**	
	Sufficient as a first test	8	9	1	2	**29**	
Attitudes towards screening	Early detection is a benefit	73	63	97	107	**403**	**928**
	Detection for better tx / more tx options	16	16	25	25	**98**	
	Sensible	42	44	54	73	**257**	
	For my general health / good	22	18	41	39	**138**	
	As part of other normal health checks	15	6	3	2	**32**	
	Want all screening / tests available	41	34	47	52	**208**	
	Benefits outweigh drawbacks of screening	84	13	72	28	**210**	
Cancer apprehension	Want to detect cancer	48	35	41	54	**213**	**485**
	Peace of mind /reassurance	46	43	66	74	**272**	
Other	General agreement	32	22	25	15	**116**	**129**
	Influence of HCP recommendation	3	1	4	4	13	
		Blood	Urine	CT	USS	Total	Theme total
		n=59	*n*=29	*n*=111	*n*=56	*n*=255	
Personal health beliefs	No family history of cancer	1	1	1	1	**5**	**50**
	Perceived good health	2	2	0	2	**8**	
	No concern for kidney cancer	2	3	3	6	**17**	
	Believe themselves to be low risk	2	1	2	6	**12**	
	Other health concerns more important	1	1	2	3	**8**	
Practicalities	Inconvenience	3	3	7	4	**20**	**43**
	Do not want to travel to hospital	2	1	10	9	**23**	
Opinions of the test	Dislike test method (general)	21	1	9	1	**33**	**124**
	Painful	3	0	0	1	**4**	
	Invasive test	3	0	0	0	**3**	
	Risks of the test	1	0	53	1	**55**	
	Would have other testing first	2	0	11	2	**15**	
	Concern regarding inaccurate results	2	2	3	5	**14**	
Attitudes towards screening	Medical care for diagnosis / treatment	4	4	7	6	**25**	**70**
	Potential to lead to further tests	1	3	1	0	**8**	
	Not a good use of NHS resources	2	2	4	2	**12**	
	Do not want any screening	4	4	4	9	**25**	
Cancer Apprehension	Worry / fear about the results	6	6	6	7	**31**	**53**
	General fear	5	2	7	6	**22**	
Other	General negative comment	4	0	5	3	**12**	**36**
	Concerns regarding medical profession	2	1	3	3	**10**	
	Beliefs about treatment of kidney cancer	4	2	4	2	**14**	

For peer review only - http://bmjopen.bmj.com/site/about/guidelines.xhtml

Table 4. Subgroup analysis: participants likely to attend screening, frequency of mention of facilitators. The colour gradients shown indicate where each value falls within the range of data shown, calculated within each subgroup. USS – ultrasound; HCP – healthcare professional; tx - treatment

Themes	Individual Codes	Participants likely to attend all screening modalities						Participants likely to attend screening by some, but not all screening modalities					
		Blood *n*=510	Urine *n*=499	CT *n*=510	USS *n*=495	Total *n*=2014	Theme total	Blood *n*=79	Urine *n*=109	CT *n*=22	USS *n*=80	Total *n*=290	Theme total
Personal Health beliefs	Life Extending / Improved survival	30	33	54	46	163	180	4	1	0	2	7	8
	Family history of cancer	2	1	2	2	7		0	0	0	1	1	
	Risk of kidney cancer	0	1	2	7	10		0	0	0	0	0	
Practicalities	Simple to complete	131	216	51	61	459	793	29	66	4	19	118	215
	Little or no inconvenience	32	64	23	25	144		6	15	3	7	31	
	Test can be conducted at GP	13	22	0	2	37		9	10	0	2	21	
	Quick	33	40	30	31	134		9	17	2	12	40	
	Cost effective	6	7	4	2	19		2	3	0	0	5	
Opinions of the test	Painless	15	21	42	46	124	541	3	5	3	11	22	133
	Test non-invasive	6	16	24	35	81		0	7	3	18	28	
	Test is without risks	16	14	22	27	79		5	9	1	19	34	
	Comfortable having test	45	16	16	19	96		7	1	1	4	13	
	Have had test before	26	12	20	36	94		8	3	0	3	14	
	Effective test	17	14	14	10	55		8	10	0	3	21	
	Low radiation	0	1	10	1	12		0	0	0	1	1	
	Sufficient as a first test	6	7	1	1	15		2	2	0	1	5	
Attitudes towards screening	Early detection is a benefit	70	61	94	98	323	734	3	2	3	9	17	47
	Detection for better tx / more tx options	15	15	25	23	78		1	1	0	2	4	
	Sensible	40	41	51	68	200		2	3	3	5	13	
	For my general health / good	21	17	41	36	115		1	1	0	3	5	
	As part of other normal health checks	9	4	3	2	18		6	2	0	0	8	
	Want all screening / tests available	41	32	45	48	166		0	2	2	4	8	
	Benefits outweigh drawbacks of screening	74	13	68	25	180		10	0	4	3	17	
Cancer apprehension	Want to detect cancer	46	32	39	47	164	383	2	3	2	7	14	24
	Peace of mind / reassurance	45	40	66	68	219		1	3	0	6	10	
Other	General agreement	27	18	23	13	81	93	5	4	2	2	13	13
	Influence of HCP recommendation	3	1	4	4	12		0	0	0	0	0	

Table 5. Subgroup analysis: participants unlikely to attend screening, frequency of mention of barriers. The colour gradients shown indicate where each value falls within the range of data shown, calculated within each subgroup. USS – ultrasound.

Themes	Individual codes	Participants unlikely to attend any screening modalities						Participants unlikely to attend screening by some, but not all screening modalities					
		Blood	Urine	CT	USS	Total	Theme total	Blood	Urine	CT	USS	Total	Theme total
		n=27	*n*=27	*n*=27	*n*=28	*n*=109		*n*=32	*n*=2	*n*=84	*n*=28	*n*=146	
Personal health beliefs	No family history of cancer	1	1	1	1	**4**	**30**	0	0	0	0	**0**	**12**
	Perceived good health	2	2	0	2	**6**		0	0	0	0	**0**	
	No concern for kidney cancer	2	3	2	2	**9**		0	0	1	4	**5**	
	Believe themselves to be low risk	1	1	0	3	**5**		1	0	2	3	**6**	
	Other health concerns more important	1	1	1	3	**6**		0	0	1	0	**1**	
Practicalities	Inconvenience	0	2	2	0	**4**	**8**	3	1	5	4	**13**	**31**
	Do not want to travel to hospital	1	1	1	1	**4**		1	0	9	8	**18**	
Opinions of the test	Dislike test method (general)	2	1	1	0	**4**	**15**	19	0	8	1	**28**	**102**
	Painful	0	0	0	0	**0**		3	0	0	1	**4**	
	Invasive test	0	0	0	0	**0**		3	0	0	0	**3**	
	Risks of the test	1	0	4	0	**5**		0	0	45	1	**46**	
	Would have other testing first	0	0	0	0	**0**		2	0	11	2	**15**	
	Concern regarding inaccurate results	1	2	1	2	**6**		1	0	2	3	**6**	
Attitudes towards screening	Medical care for diagnosis / treatment	4	4	3	4	**15**	**37**	0	0	4	2	**6**	**20**
	Potential to lead to further tests	0	2	0	0	**2**		1	1	1	0	**3**	
	Not a good use of NHS resources	1	2	1	0	**4**		1	0	3	2	**6**	
	Do not want any screening	4	4	3	5	**16**		0	0	1	4	**5**	
Cancer Apprehension	Worry / fear about the results	3	6	4	5	**18**	**26**	3	0	2	2	**7**	**19**
	General fear	3	2	1	2	**8**		2	0	6	4	**12**	
Other	General negative comment	4	0	4	2	**10**	**28**	0	0	1	1	**2**	**5**
	Concerns regarding medical profession	1	1	2	3	1		1	0	1	0	**2**	
	Beliefs about treatment of kidney cancer	4	2	3	2	4		0	0	1	0	**1**	

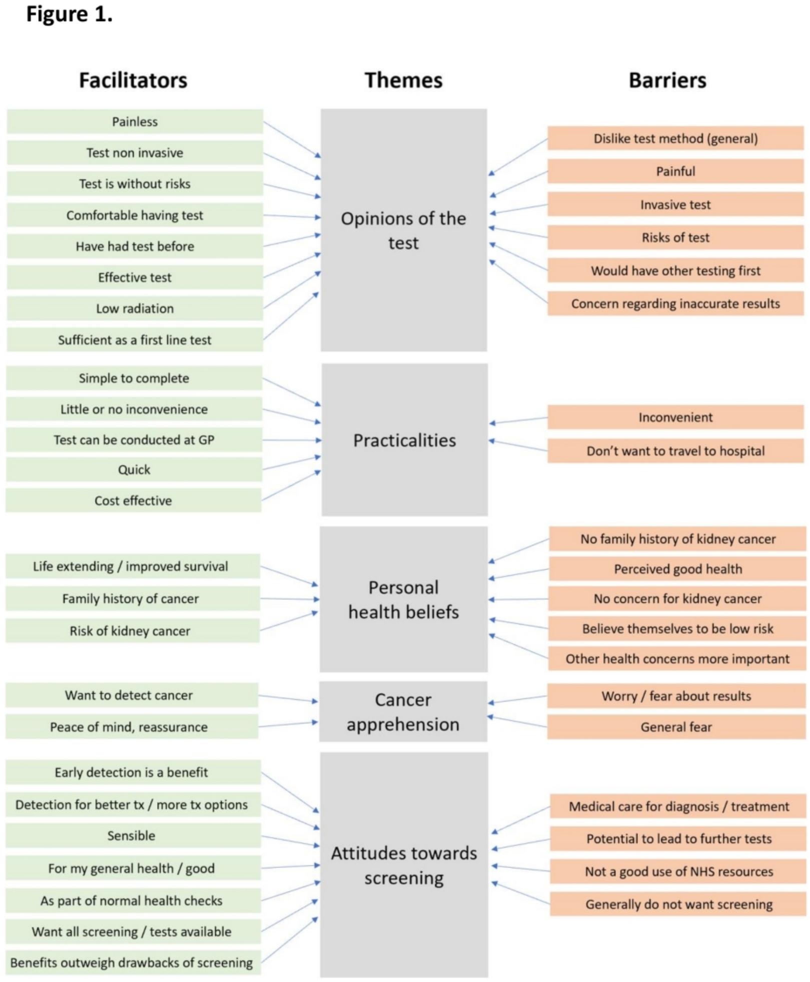

Figure 1. Individual codes (facilitators and barriers) mapped onto overarching themes

190x275mm (300 x 300 DPI)

1

Exploring attitudes towards kidney cancer screening survey

Question	Answer	Validation of question/reference
[Note that this content was delivered online so the formatting looked slightly different to in this document. The participants also did not see the headings in grey boxes or the references, but these are included to illustrate where the questions have come from.]		
Thank you for agreeing to complete this questionnaire. Please answer every question. If you are uncertain about how to answer a question, then please select the closest option.		
Demographic information<br><br>*In this first section we would like to ask you a few questions about yourself. These questions allow us to make sure we are including people from a range of different backgrounds and see if different groups of people have different views. We will not be able to identify you from your answers.*		
How old are you?	• 45-49<br>• 50-54<br>• 55-59<br>• 60-64<br>• 65-69<br>• 70-74<br>• 75-79	
What is your ethnic group? Choose one option that best describes your ethnic group or background.	• White<br>• Mixed/Multiple ethnic group<br>• Asian/Asian British<br>• Black/African/Caribbean/Black British<br>• Other, please describe______	

For peer review only - http://bmjopen.bmj.com/site/about/guidelines.xhtml

What is your highest education level?	• Finished school at or before the age of fifteen<br>• Completed CSEs, O-levels or equivalent<br>• Completed A Levels or equivalent<br>• Completed further education but not a degree<br>• Completed a Bachelor's degree / Masters' degree / PHD<br>• Other (please specify) _______	Validated from Cancer Awareness Measure (CAM) toolkit, (pre-2014, as 2014 onwards has not been validated)[1]
Are you currently:	• Employed full-time<br>• Employed part-time<br>• Unemployed<br>• Self-employed<br>• Full-time homemaker<br>• Retired<br>• Still studying<br>• Disabled or too ill to work<br>• Prefer not to say	Validated from Cancer Awareness Measure (CAM) toolkit, (pre-2014, as 2014 onwards has not been validated)[1]
Please indicate to which occupational group the Chief Income Earner in your household belongs, or which group fits best. This could be you: the Chief Income Earner is the person in your household with the largest income. If the Chief Income Earner is retired and has a pension please answer for their most recent occupation. If the Chief Income Earner is not in paid employment but has been out of work for less than 6 months, please answer for their most recent occupation.	• Professional or higher technical work - work that requires at least degree-level qualifications (e.g. doctor, accountant, schoolteacher, university lecturer, social worker, systems analyst)<br>• Manager or Senior Administrator (e.g. company director, finance	Validated method of determining social class from National Readership Survey, used by Smits et al.,2018 to give ABC1

For peer review only

	manager, personnel manager, senior sales manager, senior local government officer)<br>• Clerical (e.g. clerk, secretary)<br>• Sales or Services (e.g. commercial traveller, shop assistant, nursery nurse, care assistant, paramedic)<br>• Foreman or Supervisor of Other Workers (e.g building site foreman, supervisor of cleaning workers)<br>• Skilled Manual Work (e.g. plumber, electrician, fitter, train driver, cook, hairdresser)<br>• Semi-Skilled or Unskilled Manual Work (e.g. machine operator, assembler, postman, waitress, cleaner, labourer, driver, bar-worker, call centre worker)<br>• Other (Please specify)<br>• Have never worked	(higher SES) and C2DE (lower SES) [2, 3]<br><br>CAM toolkit survey gives examples of occupations which I think fit into the ABC1C2DE framework.[1]
**Kidney cancer screening questions - Information given about kidney cancer potential screening tests**		
Kidney cancer screening tests<br><br>*The following section is about screening for kidney cancer. You will be shown information about kidney cancer and potential screening tests and then asked your views on different screening options.*<br><br>*Kidney cancer is the 7th most common cancer in the UK. There are around 12,600 new kidney cancer cases in the UK every year and 6000 deaths from kidney cancer. In about half of the cases of kidney cancer, there are no obvious symptoms at first. These cases are often picked up during tests*		

For peer review only

carried out for other reasons. Kidney cancer can often be cured, normally by surgery, if it is caught early. However, if it is not diagnosed until it has spread beyond the kidney, then a cure is much less likely. In the UK at the moment nearly a third of all cases of kidney cancer are diagnosed after the kidney cancer had spread beyond the kidneys.

Currently there is no screening programme for kidney cancer. There is potential for a screening programme to be developed in the future though. This may help detect kidney cancer at an early stage, making it easier to treat and reducing the number of people who die from kidney cancer. As with other screening programmes, it would involve having screening tests. If these tests find anything unusual, further tests (usually a detailed CT scan) are needed to confirm a possible kidney cancer. If they find a lump in the kidney then taking a small sample of that may be recommended to confirm or rule out cancer. Some patients wish to have surgery to remove the lump without confirming it is cancer and some of these lumps will not be cancer. As such some people may have an operation that is not required and others will be worried that they have cancer when they don't. There is also a chance that a cancer could be missed as no screening test is 100% reliable. Some people will therefore be told that they don't have cancer when they do.

Over the next few pages you will see some information about each of the potential screening tests. You will then be asked how much inconvenience, burden or worry you associate with each and how likely it is that you would attend for screening with each if you were invited. Please read through the information about the tests carefully so that you can use that information to help you answer those questions.

Ultrasound scan

- *Ultrasound scanning can be used to scan the kidneys. It is similar to the scan offered to pregnant women*
- *Ultrasound scans use high frequency sound waves to make a picture of organs inside your body.*
- *You would need to go to a clinic or hospital for the scan. At the scan you would be asked to lie down and show your abdomen (tummy) by lifting your top. You would not need to undress. A cool gel would be put on your skin.*
- *The ultrasound sensor would be moved over your skin on your lower back, in order to make a picture of each kidney which will appear on a screen.*
- *Ultrasound scans are not painful and there are no risks from the scans themselves. They do not involve exposure to radiation. Most ultrasound scans last between 15 and 45 minutes*
- *If all adults in the UK were offered ultrasound screening, around 3-4 out of every 1000 people screened would have an abnormal scan and require further investigations. Approximately half of those will have kidney cancer.*

5

Low-dose computed tomography (CT) scan

- *A low-dose computed tomography (CT) scan uses x-rays to make a detailed 3 dimensional (3D) picture of the kidneys. You would need to go to a clinic or hospital for the scan. At the scan you would lie on the machine couch on your back, and the couch slowly moves through the hole of the scanner. The scanner doesn't surround your whole body at once, so you shouldn't feel claustrophobic.*
- *This type of CT scan uses no dyes, no injections, and requires nothing to swallow by mouth*
- *The actual scan takes 1-2 minutes, and the appointment would normally take around 15 minutes.*
- *CT scans are quick, painless and generally safe.*
- *You would be exposed to X-ray radiation during the scan. Generally, the amount of radiation you're exposed to during each scan is the equivalent 6 months of exposure to natural radiation from the environment. It's thought exposure to radiation during CT scans could slightly increase your chances of developing cancer many years later, although this risk is thought to be very small (less than 1 in 2,000).*
- *If all people in the UK were offered screening with CT, around 3-4 out of every 1000 people screened would have an abnormal scan and require further investigations. Approximately half of those will have kidney cancer.*

Urine sample

- *In the future we hope to be able to test for biomarkers in the urine to identify people who are likely to have kidney cancer.*
- *This approach doesn't diagnose you with kidney cancer, but it is a simple way to find out if you need further tests.*
- *This would require you to provide a urine sample in a pot and either send it off or give to your GP. Your urine sample will then be tested to see if the biomarkers are present.*
- *There are no complications from the test itself but if a test is positive for the kidney cancer biomarkers you may have to have further tests.*
- *If all people in the UK were offered screening with a urine sample, estimates are that around 2-3 out of every 1000 people screened would have an abnormal test and require further investigations. Most of those will have kidney cancer.*

For peer review only

**Blood test**		
• *In the future we hope to be able to test for biomarkers in the blood to identify people who are likely to have kidney cancer.*<br>• *This approach doesn't diagnose you with kidney cancer, but it is a simple way to find out if you need further tests.*<br>• *This would require you to have a blood test at your GP's surgery. The blood sample will tested at a laboratory to see if the biomarkers are present.*<br>• *A blood test can be slightly painful and leave a bruise.*<br>• *If the test is positive for the kidney cancer biomarkers you may have to have further tests.*<br>• *If all people in the UK were offered screening with a blood test, around 2-3 out of every 1000 people screened would have an abnormal test and require further investigations. Most of those will have kidney cancer.*		
If there was an **ultrasound based screening programme** and you were invited to have an **ultrasound scan** as part of that screening programme for kidney cancer, how likely do you think it is that you would choose to take part in the screening?	• Very likely<br>• Likely<br>• Unlikely<br>• Very unlikely	
Please describe in a few words why?	• Free text:_____	
If there was a **low-dose CT based screening programme** and you were invited to have a **low-dose CT scan** as part of a screening programme for kidney cancer, how likely do you think it is that you would choose to take part in the screening?	• Very likely<br>• Likely<br>• Unlikely<br>• Very unlikely	From Bowel cancer screening preferences survey (ongoing).
Please describe in a few words why?	• Free text:_____	
If there was a **urine sample based screening programme** and you were invited to give a **urine sample** as part of a screening programme for kidney cancer,	• Very likely<br>• Likely<br>• Unlikely	From Bowel cancer screening

For peer review only

how likely do you think it is that you would choose to take part in the screening?	• Very unlikely	preferences survey (ongoing).
Please describe in a few words why?	• Free text:_____	
If there was a **blood sample based screening programme** and you were invited to give a **blood sample** as part of a screening programme for kidney cancer, how likely do you think it is that you would choose to take part in the screening?	• Very likely<br>• Likely<br>• Unlikely<br>• Very unlikely	From Bowel cancer screening preferences survey (ongoing).
Please describe in a few words why?	• Free text:_____	
Thank you for completing the survey. Your time and responses are very much appreciated.		

References

1. Cancer Awareness Measure toolkit version 2.1 Cancer Research UK Cancer Awareness Measure (CAM) Toolkit (version 2.1). 2007. https://www.cancerresearchuk.org/sites/default/files/health_professional_cancer_awareness_measure_toolkit_version_2.1_09.02.11.pdf.

2. Smits SE, McCutchan GM, Hanson JA, Brain KE. Attitudes towards lung cancer screening in a population sample. Heal Expect. 2018; July:1–9.

3. Social Grade | National Readership Survey. http://www.nrs.co.uk/nrs-print/lifestyle-and-classification-data/social-grade/. Accessed 30 May 2019.

Consolidated criteria for reporting qualitative studies (COREQ): 32-item checklist

Developed from:
Tong A, Sainsbury P, Craig J. Consolidated criteria for reporting qualitative research (COREQ): a 32-item checklist for interviews and focus groups. *International Journal for Quality in Health Care*. 2007. Volume 19, Number 6: pp. 349 – 357

No. Item	Guide questions/description	Reported on Page #
**Domain 1: Research team and reflexivity**		
Personal Characteristics		
1. Inter viewer/facilitator	Which author/s conducted the inter view or focus group?	N/A
2. Credentials	What were the researcher's credentials? E.g. PhD, MD	8
3. Occupation	What was their occupation at the time of the study?	8
4. Gender	Was the researcher male or female?	8
5. Experience and training	What experience or training did the researcher have?	8
Relationship with participants		
6. Relationship established	Was a relationship established prior to study commencement?	N/A
7. Participant knowledge of the interviewer	What did the participants know about the researcher? e.g. personal goals, reasons for doing the research	N/A
8. Interviewer characteristics	What characteristics were reported about the inter viewer/facilitator? e.g. Bias, assumptions, reasons and interests in the research topic	N/A
**Domain 2: study design**		
Theoretical framework		
9. Methodological orientation and Theory	What methodological orientation was stated to underpin the study? e.g. grounded theory, discourse analysis, ethnography, phenomenology, content analysis	
Participant selection		
10. Sampling	How were participants selected? e.g. purposive, convenience, consecutive, snowball	7
11. Method of approach	How were participants approached? e.g. face-to-face, telephone, mail, email	7
12. Sample size	How many participants were in the study?	7
13. Non-participation	How many people refused to participate or dropped out? Reasons?	7
Setting		

For peer review only

14. Setting of data collection	Where was the data collected? e.g. home, clinic, workplace	5
15. Presence of non-participants	Was anyone else present besides the participants and researchers?	N/A
16. Description of sample	What are the important characteristics of the sample? e.g. demographic data, date	Table 1
Data collection		
17. Interview guide	Were questions, prompts, guides provided by the authors? Was it pilot tested?	7 and Supplementary file 1
18. Repeat interviews	Were repeat inter views carried out? If yes, how many?	N/A
19. Audio/visual recording	Did the research use audio or visual recording to collect the data?	N/A
20. Field notes	Were field notes made during and/or after the inter view or focus group?	N/A
21. Duration	What was the duration of the inter views or focus group?	N/A
22. Data saturation	Was data saturation discussed?	N/A
23. Transcripts returned	Were transcripts returned to participants for comment and/or correction?	N/A
**Domain 3: analysis and findings**		
Data analysis		
24. Number of data coders	How many data coders coded the data?	8
25. Description of the coding tree	Did authors provide a description of the coding tree?	Figure 1
26. Derivation of themes	Were themes identified in advance or derived from the data?	8
27. Software	What software, if applicable, was used to manage the data?	9
28. Participant checking	Did participants provide feedback on the findings?	N/A
Reporting		
29. Quotations presented	Were participant quotations presented to illustrate the themes/findings? Was each quotation identified? e.g. participant number	Table 2
30. Data and findings consistent	Was there consistency between the data presented and the findings?	Results
31. Clarity of major themes	Were major themes clearly presented in the findings?	11-14
32. Clarity of minor themes	Is there a description of diverse cases or discussion of minor themes?	11-14

For peer review only

BMJ Open

Reasons for intending to accept or decline kidney cancer screening: thematic analysis of free text from an online survey

Journal:	*BMJ Open*
Manuscript ID	bmjopen-2020-044961.R1
Article Type:	Original research
Date Submitted by the Author:	17-Mar-2021

Complete List of Authors:	Freer-Smith, Charlotte; University of Cambridge School of Clinical Medicine<br>Harvey-Kelly, Laragh ; University of Cambridge School of Clinical Medicine<br>Mills, Katie; University of Cambridge, Department of Public Health and Primary Care<br>Harrison, Hannah; University of Cambridge, The Primary Care Unit, Department of Public Health and Primary Care<br>Rossi, Sabrina; University of Cambridge, Department of Surgery; Cambridge University Hospitals NHS Foundation Trust, Department of Urology<br>Griffin, Simon; University of Cambridge, Department of Public Health and Primary Care<br>Stewart, Grant; University of Cambridge, ;<br>Usher-Smith, Juliet; University of Cambridge, Department of Public Health and Primary Care

**Primary Subject Heading**:	Public health
Secondary Subject Heading:	Oncology
Keywords:	Kidney tumours < ONCOLOGY, PREVENTIVE MEDICINE, PUBLIC HEALTH, QUALITATIVE RESEARCH

SCHOLARONE™
Manuscripts

I, the Submitting Author has the right to grant and does grant on behalf of all authors of the Work (as defined in the below author licence), an exclusive licence and/or a non-exclusive licence for contributions from authors who are: i) UK Crown employees; ii) where BMJ has agreed a CC-BY licence shall apply, and/or iii) in accordance with the terms applicable for US Federal Government officers or employees acting as part of their official duties; on a worldwide, perpetual, irrevocable, royalty-free basis to BMJ Publishing Group Ltd ("BMJ") its licensees and where the relevant Journal is co-owned by BMJ to the co-owners of the Journal, to publish the Work in this journal and any other BMJ products and to exploit all rights, as set out in our licence.

The Submitting Author accepts and understands that any supply made under these terms is made by BMJ to the Submitting Author unless you are acting as an employee on behalf of your employer or a postgraduate student of an affiliated institution which is paying any applicable article publishing charge ("APC") for Open Access articles. Where the Submitting Author wishes to make the Work available on an Open Access basis (and intends to pay the relevant APC), the terms of reuse of such Open Access shall be governed by a Creative Commons licence – details of these licences and which Creative Commons licence will apply to this Work are set out in our licence referred to above.

Other than as permitted in any relevant BMJ Author's Self Archiving Policies, I confirm this Work has not been accepted for publication elsewhere, is not being considered for publication elsewhere and does not duplicate material already published. I confirm all authors consent to publication of this Work and authorise the granting of this licence.

For peer review only

Reasons for intending to accept or decline kidney cancer screening: thematic analysis of free text from an online survey

Charlotte Freer-Smith[a], Laragh L.W. Harvey-Kelly[a], Katie Mills[b], Hannah Harrison[b], Sabrina H. Rossi[c], Simon J. Griffin [b], Grant D. Stewart[d], Juliet A. Usher-Smith[b]

[a] University of Cambridge School of Clinical Medicine, Addenbrooke's Hospital, Hills Road, Cambridge, CB2 0SP, UK

[b] The Primary Care Unit, Department of Public Health and Primary Care, University of Cambridge, Cambridge, CB2 0SR, UK

[c] Department of Oncology, University of Cambridge, Addenbrooke's Hospital, Cambridge Biomedical Campus, Hills Road, Cambridge CB2 0QQ, UK

[d] Department of Surgery, University of Cambridge, Addenbrooke's Hospital, Cambridge Biomedical Campus, Hills Road, Cambridge CB2 0QQ, UK

Corresponding author:

Dr Juliet Usher-Smith

The Primary Care Unit,

Department of Public Health and Primary Care,

University of Cambridge, Cambridge, CB2 0SR, UK

Tel: 01223 748693

Email: jau20@medschl.cam.ac.uk

Word count: 3998

For peer review only - http://bmjopen.bmj.com/site/about/guidelines.xhtml

For peer review only

ABSTRACT

Objectives: Kidney cancer has been identified as a disease for which screening might provide significant benefit for patients. The aim of this study was to understand in detail the facilitators and barriers towards up-take of a future kidney cancer screening programme, and to compare these across four proposed screening modalities.

Design: An online survey including free text responses.

Setting: UK

Participants: 668 adults

Primary and secondary outcome measures: The survey assessed participants' self-reported intention to take-up kidney cancer screening with four different test methods (urine test, blood test, ultrasound scan, low-dose CT). We conducted thematic analysis of 2559 free text comments made within the survey using an inductive approach.

Results: We identified five overarching themes that influenced screening intention: 'personal health beliefs', 'practicalities', 'opinions of the test', 'attitudes towards screening' and 'cancer apprehension'. Overall participants considered the tests presented as simple to complete and the benefits of early detection to outweigh any drawbacks to screening. Dominant facilitators and barriers varied with patterns of intention to take up screening across the four tests. Most intended to take up screening by all four tests, and for these participants, screening was seen as a positive health behaviour. A significant minority were driven by practicalities and the risks of the tests offered. A smaller proportion intended to reject all forms of screening offered, often due to fear or worry about results and unnecessary medical intervention or a general negative view of screening.

For peer review only - http://bmjopen.bmj.com/site/about/guidelines.xhtml

For peer review only

Conclusions: Most individuals would accept kidney cancer screening by any of the four test options presented because of strong positive attitudes towards screening in general and the perceived simplicity of the tests. Providing information about the rationale for screening in general and the potential benefits of early detection will be important to optimise uptake amongst uncertain individuals.

Key words: Kidney cancer; Cancer; Screening; Survey; Attitudes; Public opinions

For peer review only

Strengths and Limitations of this study

- The free text nature of the questions allowed participants to provide their own reasoning without being prompted or constrained by a list of predefined options
- The large sample size meant that we were able to explore reasons for both intending to take up screening and for not taking up screening
- Our use of an online recruitment platform means the views of those who completed our survey may not be representative of the general population
- It also meant we relied on written comments from participants and were unable to explore their comments or views in-depth
- In the absence of an existing screening programme we were also only able to assess reasons behind intention and not attendance at screening

For peer review only

For peer review only - http://bmjopen.bmj.com/site/about/guidelines.xhtml

INTRODUCTION

Kidney cancer is the ninth most common cancer worldwide in men and the fourteenth most common cancer in women[1], with over 400,000 new cases being diagnosed in 2018[2]. The incidence of kidney cancer in the UK is projected to rise by 26% between 2014 and 2035, representing one of the fastest accelerating cancers within that timeframe[3]. Nearly a quarter of individuals diagnosed with kidney cancer in the UK have evidence of metastatic disease at presentation[4]; the five year age-adjusted relative survival is 86% for those diagnosed with the earliest stage of the disease, but drops to 12% for metastatic disease. These epidemiologic data have led to increasing interest in screening for kidney cancer, with the development of a suitable screening programme being identified as a key research priority[5].

For a screening programme to be successful, it must be acceptable to the general public and uptake must be high. Previous research in the context of other screening programmes has identified a number of factors influencing screening uptake. These include practical barriers, for example, difficulty making an appointment or lack of time to do so[6,7], as well as psycho-social barriers such as fear or embarrassment[7–9]. Identified facilitators of screening attendance encompass both societal factors such as public education, physician recommendation and social networks[10,11], and personal factors such as positive attitudes towards screening[12], the desire for peace of mind[9,13], and subjective assessments of individual health status and cancer risk[11,14]. However, many of these existing studies have identified public opinion through qualitative methods with small sample sizes, or via surveys using pre-defined lists of possible responses. Furthermore, these studies have focused on cancers for which there are existing national screening programmes (breast, cervical and

For peer review only - http://bmjopen.bmj.com/site/about/guidelines.xhtml

colorectal cancer). Compared to these cancers, kidney cancer has a lower prevalence[3], public awareness of the condition is lower[15], and the range of potential screening modalities is greater. Understanding the specific reasons why people intend to take up or decline kidney cancer screening with different screening tests is therefore important when developing future kidney cancer screening programmes

In an online survey we have shown that a future kidney cancer screening programme would be positively received and intention to attend high, whilst the choice of screening test is likely to have an important influence on uptake[16] . The aim of the current study was to use free text responses to questions in the online survey to describe the reasons for intending to accept or decline kidney cancer screening with four different putative screening test options (urine dipstick analysis, blood based biomarkers, low dose computed tomography (CT) and ultrasound). The free text responses are used to understand in detail the reasons for those findings, identify the specific barriers and facilitators for kidney cancer screening, and to compare these drivers across four proposed screening modalities.

METHODS

Study design

This study reports analysis of free text answers from an online population-based survey of 668 members of the public in the UK aged 45-77 years.

Participants and recruitment

Participants were recruited to complete the survey through an online participant recruitment platform for researchers (Prolific, www.prolific.ac). Individuals registered with

For peer review only - http://bmjopen.bmj.com/site/about/guidelines.xhtml

Prolific were eligible to take part if they were between 45-79 years old and had a Prolific approval rating ≥95% (reflecting the percentage of studies completed that have been approved by researchers). The survey was added to the Prolific platform on 20/08/2019. At that time, 7,767 met the eligibility criteria for the study. Based on demographic data available on the Prolific website, approximately 80% of these were White/Caucasian, 34% male, 53% lived in the UK, 15% were current smokers, 43% had a university level education and 51% considered themselves to be in the top five deciles of socioeconomic status. The survey was live for eight hours. During this time 1,190 participants viewed the participant information leaflet and consent form. A total of 1,021 participants completed the survey. The analysis reported here is based on the free-text comments from the 668 UK based participants. We chose to limit to UK participants for this analysis to remove any differences in views arising from cultural differences in attitudes towards cancer, screening and different healthcare or insurance systems.

Survey

A copy of the relevant sections of the survey can be found in Supplementary file 1. In brief, after providing basic demographic data, participants were presented with an overview of kidney cancer, and then the details of four potential screening methods (urine testing, blood biomarkers, low-dose CT and ultrasound). This information included the number of people likely to test positive with each test method, and of those, how many people would actually have cancer. These summaries were developed in collaboration with patient and public representatives, based on the best available evidence, and the information was reviewed by two urologists within the research team (GDS, SHR). Following presentation of the details of

For peer review only - http://bmjopen.bmj.com/site/about/guidelines.xhtml

the screening methods, participants were asked how likely they would be to take part in screening with each method (response options were 'Very likely', 'Likely', 'Unlikely' and 'Very unlikely'). Participants were subsequently asked to "*describe in a few words why*?" they had given that response for each screening modality. This analysis focuses on the free-text responses to those four open-ended questions.

Qualitative Analysis

We conducted thematic analysis of all free text comments using an inductive approach, with initial coding of responses being driven by the content of the comments themselves. Responses were analysed in two groups based on whether participants responded positively ('likely' or 'very likely' to take part) or negatively ('unlikely' or 'very unlikely' to take part). From this point, these groups will be referred to as 'likely' or 'unlikely' respectively. During the analysis process, comments that were inconsistent with the participant's previous answer were removed (e.g. if they answered 'very unlikely' to take part but provided a free text answer supporting screening). Incomplete data (where participants did not provide any free text) were also removed.

Two researchers (KM, a researcher with qualitative expertise and CFS a medical student with a qualitative research background) familiarised themselves with a random sample of 400 comments, representing 15% of the total data and equally split between those likely or unlikely to take part, and developed initial codes independently. They then discussed and reviewed the coding decisions and developed two consolidated lists: one for the 'likely' to take part comments, and one for the 'unlikely' to take part comments (Figure 1). The remaining free text comments were coded into these agreed lists within an excel file by one

For peer review only - http://bmjopen.bmj.com/site/about/guidelines.xhtml

For peer review only

researcher (CFS). If a comment contained multiple meanings, the comment was assigned to as many codes as appropriate to cover the content. At this stage, 5% of the total sample, selected at random, was coded by two researchers (CFS and LHK) and any discrepancies discussed. Agreement between the two was 94%. The full list of codes was then discussed at a meeting with the entire research team. Within this meeting, the group mapped the individual codes into overarching themes.

To compare the relative importance of individual codes and themes, the number of times a code was mentioned for each of the four screening tests was counted. We used this data first to explore differences in responses between screening modalities, descriptively highlighting the differences in code frequencies. We then divided participants three subgroups: those likely to take up screening by all four test methods, those unlikely to take up screening by any of the methods, and those whose intention varied between the tests. Differences in the patterns of responses between these groups were explored. Finally, we investigated the patterns of responses with sex, age, level of education, and social group.

Ethics approval and consent to participate

Ethical approval was obtained from the Psychology Research Ethics committee of the University of Cambridge (Ref 2019.055). All participants gave informed written online consent before they began the survey.

Patient and Public involvement

For peer review only - http://bmjopen.bmj.com/site/about/guidelines.xhtml

Two members of the public contributed, through face-to-face discussions and email, to the development of the survey and participant information sheet. They also provided comments on the final manuscript prior to submission.

RESULTS

The sociodemographic profile of the sample is shown in Table 1. 373 (54%) were female, with a mean age of 54.7±7 years and a good distribution across age groups. Most (73%) participants were in social group ABC1 (those with managerial, administrative, or professional job roles) and 288 (43.1%) had university level education.

Intention to take up kidney cancer screening

Overall, the majority of participants (518, 78%) were likely to take up screening by all four screening test modalities. Of the remaining participants, 119 (18%) would be likely to take up some screening tests but not all, with 55/119 (46%) likely to attend for an ultrasound, urine or blood testing, but not for a low-dose CT scan. 31 (5%) indicated that they were unlikely to take up screening by any of the test methods. Since intention to take-up screening was high, we collected a greater number of free-text comments relating to reasons why someone was likely to take part in screening. Once inconsistent and blank data had been removed (51 and 62 participants respectively) there were 2304 comments from those ‘likely’ to take up screening and 255 comments from those ‘unlikely’ to take up a screening.

Themes identified

For peer review only - http://bmjopen.bmj.com/site/about/guidelines.xhtml

From the free text comments, we identified 49 individual codes (27 facilitators and 22 barriers from those likely and unlikely to take up screening respectively). On average, each comment was coded under 1.5 codes: 1,448 (56.6%) of comments were relevant to one code, 907 (35.4%) were relevant to two codes, with the remaining 204 (8%) relevant to 3 or more. Together the codes mapped onto five overarching themes (Figure 1): Personal health beliefs; cancer apprehension; attitudes towards screening; opinions of the test; and practicalities (additionally, we included an 'other' category containing five codes that did not map elsewhere).. Illustrative quotes from each theme are presented in Table 2.

Facilitators and barriers identified across screening methods

Table 3 shows the frequency with which each of the facilitators and barriers were reported, overall and across the four screening modalities. 648 participants provided comments relating to blood tests. 589 (91%) of those were likely to attend. Comments amongst that group focused primarily on statements that blood testing is '*simple to complete*' (160, 25%), the '*benefits of screening outweigh the drawbacks*' (84, 13%) and '*early detection is a benefit*' (73, 11%). Of those unlikely to take up blood testing (59, 9%), the only consistently mentioned barrier was a '*dislike of the test method'*, mentioned by 3% (21).

Among the 637 participants providing comments on urine testing, 608 (95%) were likely to take up screening. Similar to the responses for blood testing, the most common overall reason for taking up screening was that the test is '*simple to complete'* (282, 44%). Frequently cited other comments that related specifically to the characteristics of the test were '*little or no inconvenience'* (79, 12%) and '*quick'* (57, 9%). *'Early detection is a benefit'* was also frequently mentioned by this group (63, 10%) but references to *'benefits of*

For peer review only - http://bmjopen.bmj.com/site/about/guidelines.xhtml

screening outweigh the drawbacks' were less common, potentially reflecting that fewer drawbacks are associated with urine testing than blood testing. Overall, no single barrier to urine testing was identified, with no code occurring more than 6 times.

631 participants provided comments relating to ultrasound scans and 643 relating to CT. Intention to take up screening was again high with 575 (91%) likely to attend ultrasound and 532 (82%) CT. Notably, among participants likely to accept screening by ultrasound or CT the most mentioned facilitator for both does not relate to the test itself but the belief that *'early detection is a benefit'*, (97 (15%) for CT scan, 107 (17%) for ultrasound). Although less dominant, *'simple to complete'* was still mentioned for both (55 (9%) for CT, 80 (13%) for ultrasound). For ultrasound, there are also multiple codes within the theme 'opinions of the test' that are mentioned more frequently than for other test methods: *'painless'* (57, 9%) '*non-invasive'* (53, 8%) and *'without risks'* (46, 7%). Of these facilitators, only *'painless'* is mentioned with some frequency for CT scans (45, 7%). As seen for blood tests, *'benefits of screening outweigh the drawbacks'* is also a dominant facilitator for CT scans.

The key barrier identified by those unlikely to take up a CT scan is the *'risk of the test'* (53, 8%), some mentioning only concerns regarding the danger of radiation, while others linked this to long term cancer risks. An additional barrier highlighted for both CT and ultrasound, but not blood or urine testing, was travel to a hospital (10 (2%) and 9 (1%) respectively).

Notably, across all test modalities and across those likely or unlikely to attend screening, there were very few comments that related to the risk of kidney cancer specifically or the accuracy or relative costs of the tests. Evaluation of the responses by demographics also revealed no clear patterns with age, sex, social group or university education.

For peer review only - http://bmjopen.bmj.com/site/about/guidelines.xhtml

Subgroup thematic analysis

Comparison of the comments made by participants who said they were likely to take up screening by all four test methods with those who would accept screening by some of the test methods only is presented in Table 4. For those whose likelihood of taking up screening varied between the different tests, 'practicalities' and 'opinions of the test' remained the most prominent themes. *'Simple to complete'* represented the most frequently mentioned individual code (41%, n=118/290 comments), with most of these from comments in relation to urine or blood testing. The non-invasive nature of ultrasound scanning, and perceived lack of risks associated with this test were also seen in this group.

Conversely, for participants who would accept screening with all four tests, the themes 'attitudes towards screening', 'cancer apprehension' and 'personal health beliefs' are proportionally more important. Whilst *'simple to complete'* is still the most mentioned individual code, others within these additional themes become equally important. The facilitators identified within the 'attitudes towards screening' theme reflect a general attitude that screening is a "correct" form of behaviour: *'sensible'* (10%, n=200/2014 comments), *'for my general health / good'* (6%, n=115/2014), *'early detection is a benefit'* (16%, n=323/2014 comments). The perceived benefits of early detection that were highlighted by participants included improved survival, better treatment, and more treatment options. Many participants, however, simply stated that early detection is always beneficial and did not give further explanation. Another common belief described by this group was the idea that screening could provide a sense of *'peace of mind / reassurance'* that they did not have the disease (11%, n=219/2014), highlighting one way in which cancer worry can motivate screening attendance.

For peer review only - http://bmjopen.bmj.com/site/about/guidelines.xhtml

Table 5 shows the barriers cited by those who are unlikely to take up screening by any of the tests and those whose attitude towards screening varied with screening modality. For those declining some test methods, but not all, the only dominant theme is 'opinions of the test': most of these comments reference the *'risks of the test'* for CT scan (54%, 45/84), or a *'dislike of the test'* for blood tests (59%, 19/32). Very few other barriers outside of these are mentioned. In contrast, those who decline all screening methods do not mention test specific concerns, instead 'attitudes towards screening', 'personal health beliefs' and 'cancer apprehension' are all more frequently raised across all the test modalities. The most frequently mentioned individual barrier to screening is *'worry / fear about results'* (17%, 18/109). Many also state that they '*do not want any screening'* (15%, 16/109) or they believe that that '*medical care is for diagnosis / symptoms only'* (14%, 15/109), reflecting a disagreement with the principle of screening in general.

DISCUSSION

Key Findings

Through a thematic analysis of over 2000 free text comments from an online survey, we have identified, and ranked the relative importance of, facilitators and barriers influencing potential uptake of kidney cancer screening by four possible screening modalities in a UK population. We found that, overall, across all four tests most participants considered screening simple to complete and that the benefits of early detection outweigh any burdens or harms. Urine testing was viewed as the simplest and least invasive to complete; without the risks or inconvenience associated with other tests. Additionally, we found that the dominant drivers and barriers varied with patterns of intention to take up screening across

For peer review only - http://bmjopen.bmj.com/site/about/guidelines.xhtml

the four tests. The most frequent pattern, and therefore the largest group of individuals, was those who were likely to take up screening with all four tests. Amongst this group, screening was seen as a positive health behaviour, with many benefits: the tests are seen as simple, they see early detection as an advantage, or seek the peace of mind and clarity that accompanies a negative screening result. The second group was those for whom the specific test modality influences decision making. Within this group a significant minority would attend all tests except for a CT scan, due to concern about the risk associated with a CT. Blood tests also polarised participants: most were comfortable with this method and it was seen as simple by many, but a small minority expressed a specific dislike of blood tests. Having to travel to hospital for a CT or USS also put some people off these modalities. The smallest group was those who reject all forms of screening. For these individuals, the drawbacks of screening in general are highlighted rather than opinions related to specific tests. These drawbacks include fear or worry about results and unnecessary medical intervention and are not balanced by sufficient benefits. Notably, largely absent from the reasons that participants did or did not intend to take up screening among any of these groups were comments related to the accuracy of the tests or the risk of kidney cancer specifically, despite information on both of these being included within the survey.

Comparison with existing literature

Our finding that the decision to take up screening in this study for many of the participants was driven by the perceived value of early detection, with screening embodying a positive health behaviour (or a sensible decision) is consistent with previous studies exploring facilitators of screening in existing cancer screening programmes[10,11,13,17–19] and the view

For peer review only - http://bmjopen.bmj.com/site/about/guidelines.xhtml

that screening is 'almost always a good idea'[20]. Our study builds on these findings by showing that these beliefs about screening extend to hypothetical new screening programmes and, for the individuals who hold these views, the nature of the screening test is not a key factor in their decision of whether to take up screening or not. This observation, that screening decisions are often driven by emotions and strongly held contextual beliefs, was also seen among those who did not intend to attend screening with any of the tests. While a small number of participants in this group appear to have been consciously weighing up the potential benefits and harms of kidney cancer screening within the context of their own personal health beliefs, our findings suggest many may be driven by a more general aversion to the concept of screening or apprehension about cancer in general or the results. This has been reported in the context of other screening programmes[12,21–25] and highlights the need for information materials to clarify the purpose of screening and advantages of early diagnosis in asymptomatic individuals[22] and to specifically address negative beliefs around the disease and cancer worry. The preference for simple and convenient tests, seen through the responses for urine and blood testing in particular in this study, has also been reported previously in the context of other cancer screening programmes[7,14,26]. Our findings additionally show that the risks associated with exposure to radiation for CT are a key barrier for some individuals.

Strengths and Limitations

The main strengths of this study are the large sample size and the free text nature of the questions that allowed participants to provide their own reasoning without being prompted by a list of predefined options.

For peer review only - http://bmjopen.bmj.com/site/about/guidelines.xhtml

The large sample size meant that we were able to explore reasons for not taking up screening among the minority of participants who would be unlikely to take it up. Uptake rates for UK cancer screening programmes are consistently below 75%[27–29], therefore, capturing the perspectives of those who are less likely to accept screening (and difficult to engage in research) is important. Since we were also only able to assess the reasoning behind the participants' intention to attend screening, and not their actual attendance, this highlights a limitation of our study, as intention may be higher than attendance. We also relied on the written comments and were unable to go back to individuals for clarification or further in-depth exploration of their views.

As a result of our use of an online recruitment method the views of those who completed our survey may also not be representative of the general population[30]. In particular, the distribution across social groups was different: 73% of our participants reported being in the upper half of the social grades (ABC1), whereas census data from 2011 suggests 53% of the UK population lies within this category. Given that participation in screening programmes is disproportionately lower among lower social grades[19,31], the participants in our study may, therefore, be more enthusiastic about screening than the UK population as a whole. National screening programmes for cancer are also established within the UK and provided at no cost to participants within a state funded healthcare system. Awareness of these government programmes and the absence of cost as a barrier may have influenced the participants views. There are also known cultural variations in attitudes towards cancer and awareness of cancer risk that may influence response to screening[32,33]. While we would expect the range of attitudes to be similar in other countries, the relative frequency of the individual codes and themes may therefore differ. With online data collection there is also the risk that participants did not concentrate and their responses do not accurately reflect

For peer review only - http://bmjopen.bmj.com/site/about/guidelines.xhtml

For peer review only

their views. We sought to limit this by including an instructional manipulation check early on the questionnaire. Only 51 of the 2,559 comments (1.9%) were also inconsistent, suggesting that the overwhelming majority of participants were appropriately engaged with the survey.

The free text nature of the questions meant that participants were free to express their thoughts in their own words. This has the advantage of not constraining them by pre-defined lists. However, the question we asked ("*Please describe in a few words why*?") did not instruct participants to list all the reasons behind their choice, meaning responses ranged from one word to several sentences. Our findings, therefore, reflect the dominant views of participants when faced with the option of a given screening test, not all their views. The relative frequency of the different codes and themes reflects their importance and not the necessarily the number of participants for whom that factor may contribute to their decision.

CONCLUSIONS

Our findings show that most individuals in a UK population think they are likely to attend kidney cancer screening by any of the four test options presented. These individuals are driven both by a general perception of screening as a good thing and the view that the tests are simple, making them likely to participate in any future kidney cancer screening programme largely without consideration of the nature of the test involved or their own risk of kidney cancer. For the significant minority for whom the practicalities and risks of the tests are important, optimising the logistics of the screening programme and ensuring that the written information is understandable, for example around the radiation risk associated with CT, will be important. Providing sufficient information to enable individuals to make an

For peer review only - http://bmjopen.bmj.com/site/about/guidelines.xhtml

informed choice about screening will also be important for the small proportion of the population who do not intend to take up screening with any of the test options. Our findings suggest that that information should not be limited to the specific details of kidney cancer screening but should also include an explanation of the rationale for screening in general and the potential benefits of early detection.

Acknowledgements

We would like to thank our two patient and public representatives Phil Alsop and Philip Dondi for their input and advice throughout this study.

Authors' contributions

JUS, LHK, KM, HH, SHR, GDS and SG were involved in the design of the study. JUS completed data collection. CFS, KM, LHK were involved in initial data analysis, and all authors contributed to the final analysis and interpretation of the data. CFS and JUS wrote the first draft of the manuscript. All authors critically reviewed the manuscript and have approved the final version.

Funding

This work was funded by a research grant from Kidney Cancer UK (no award number). JUS and KM were supported by a Cancer Research UK Cancer Prevention Fellowship (C55650/A21464). SHR is supported by The Urology Foundation (no award number) and a

For peer review only - http://bmjopen.bmj.com/site/about/guidelines.xhtml

For peer review only

Cancer Research UK Clinical Research Fellowship (no award number). GDS is funded by the Cancer Research UK Cambridge Cancer Centre (Major Centre Award A25117) and the Renal Cancer Research Fund (no award number). HH is supported by a National Institute of Health Research Methods Fellowship (RM-SR-2017-09-009). The University of Cambridge has received salary support in respect of SJG from the NHS in the East of England through the Clinical Academic Reserve.

The views expressed in this publication are those of the author(s) and not necessarily those of the NHS, the National Institute for Health Research or the Department of Health and Social Care

Data sharing

The dataset supporting the conclusions of this article will be made available in the University of Cambridge data repository (https://www.repository.cam.ac.uk/) for at least 10 years from the last access. All the data will be stored in accordance with the Data Protection Act 1998.

Financial disclosure: All authors have completed the Unified Competing Interest form at www.icmje.org/coi_disclosure.pdf (available on request from the corresponding author). GDS has received educational grants from Pfizer, AstraZeneca and Intuitive Surgical; consultancy fees from Pfizer, Merck, EUSA Pharma and CMR Surgical; Travel expenses from Pfizer and Speaker fees from Pfizer. All other authors declare that (1) they have no support from or relationships with companies that might have an interest in the submitted work in

For peer review only

the previous 3 years; (2) their spouses, partners, or children have no financial relationships that may be relevant to the submitted work; and (3) they have no non-financial interests that may be relevant to the submitted work.

The corresponding author has the right to grant on behalf of all authors and does grant on behalf of all authors, an exclusive licence on a worldwide basis to the BMJ Publishing Group Ltd and its Licensees to permit this article (if accepted) to be published in BMJ editions and any other BMJPGL products and sub-licences to exploit all subsidiary rights, as set out in our licence (http://resources.bmj.com/bmj/authors/checklists-forms/licence-for-publication).

All authors had full access to all of the data in the study and can take responsibility for the integrity of the data and the accuracy of the data analysis

The corresponding author affirms that the manuscript is an honest, accurate, and transparent account of the study being reported; that no important aspects of the study have been omitted; and that any discrepancies from the study as planned (and, if relevant, registered) have been explained.

For peer review only - http://bmjopen.bmj.com/site/about/guidelines.xhtml

REFERENCES

1 Znaor A, Lortet-Tieulent J, Laversanne M, *et al.* International Variations and Trends in Renal Cell Carcinoma Incidence and Mortality. *Eur Urol* 2015;**67**:519–30. doi:10.1016/j.eururo.2014.10.002

2 Bray F, Ferlay J, Soerjomataram I, *et al.* Global cancer statistics 2018: GLOBOCAN estimates of incidence and mortality worldwide for 36 cancers in 185 countries. *CA Cancer J Clin* 2018;**68**:394–424. doi:10.3322/caac.21492

3 Smittenaar CR, Petersen KA, Stewart K, *et al.* Cancer incidence and mortality projections in the UK until 2035. *Br J Cancer* 2016;**115**:1147–55. doi:10.1038/bjc.2016.304

4 Cancer survival in England - Office for National Statistics. https://www.ons.gov.uk/peoplepopulationandcommunity/healthandsocialcare/conditionsanddiseases/bulletins/cancersurvivalinengland/nationalestimatesforpatientsfollowedupto2017#:~:text=Cancer survival in children (aged,year and 10-year survival. (accessed 10 Jul 2020).

5 Rossi SH, Fielding A, Blick C, *et al.* Setting Research Priorities in Partnership with Patients to Provide Patient-centred Urological Cancer Care. Eur. Urol. 2019;**75**:891–3. doi:10.1016/j.eururo.2019.03.008

6 Waller J, Bartoszek M, Marlow L, *et al.* Barriers to cervical cancer screening attendance in England: a population-based survey. 2009;**16**:199–204.

7 Aubin-Auger I, Mercier A, Lebeau J-P, *et al.* Obstacles to colorectal screening in general practice: a qualitative study of GPs and patients. *Fam Pract* 2011;**28**:670–6.

For peer review only - http://bmjopen.bmj.com/site/about/guidelines.xhtml

doi:10.1093/fampra/cmr020

8 Bukowska-Durawa A, Luszczynska A. Cervical cancer screening and psychosocial barriers perceived by patients. A systematic review. Wspolczesna Onkol. 2014;**18**:153–9. doi:10.5114/wo.2014.43158

9 Ritvo P, Myers RE, Paszat L, *et al.* Gender differences in attitudes impeding colorectal cancer screening. *BMC Public Health* 2013;**13**:1. doi:10.1186/1471-2458-13-500

10 Young B, Bedford L, Kendrick D, *et al.* Factors influencing the decision to attend screening for cancer in the UK: a meta-ethnography of qualitative research. *J Public Health (Bangkok)* 2017;**40**:315–39. doi:10.1093/pubmed/fdx026

11 Honein-abouhaidar GN, Kastner M, Vuong V, *et al.* Systematic Review and Meta-study Synthesis of Qualitative Studies Evaluating Facilitators and Barriers to Participation in Colorectal Cancer Screening. 2016;:907–18. doi:10.1158/1055-9965.EPI-15-0990

12 Smits SE, McCutchan GM, Hanson JA, *et al.* Attitudes towards lung cancer screening in a population sample. *Heal Expect* 2018;:1–9. doi:10.1111/hex.12819

13 Robb KA, Solarin I, Power E, *et al.* Attitudes to colorectal cancer screening among ethnic minority groups in the UK. *BMC Public Health* 2008;**8**. doi:10.1186/1471-2458-8-34

14 Edgar L, Glackin M, Hughes C, *et al.* Factors influencing participation in breast cancer screening. *Br J Nurs* 2013;**22**:1021–6.

15 Public awareness of cancer in Britain: Report for the National Awareness and Early Diagnosis Initiative. 2009 Opinions Survey. https://www.cancerresearchuk.org/sites/default/files/public_awareness_of_cancer_i

For peer review only - http://bmjopen.bmj.com/site/about/guidelines.xhtml

n_britain_dh_report.pdf

16 Harvey-Kelly LLW, Harrison H, Rossi SH, *et al.* Public attitudes towards screening for kidney cancer: an online survey. *BMC Urol* 2020;:1–10. doi:10.1186/s12894-020-00724-0

17 Dam L Van, Korfage IJ, Kuipers EJ, *et al.* What influences the decision to participate in colorectal cancer screening with faecal occult blood testing and sigmoidoscopy ? *Eur J Cancer* 2013;**49**:2321–30. doi:10.1016/j.ejca.2013.03.007

18 James LJ, Wong G, Craig JC, *et al.* Men's perspectives of prostate cancer screening: A systematic review of qualitative studies. *PLoS One* 2017;**12**:e0188258. doi:10.1371/journal.pone.0188258

19 Pfeffer N. Screening for breast cancer: Candidacy and compliance. *Soc Sci Med* 2004;**58**:151–60. doi:10.1016/S0277-9536(03)00156-4

20 Waller J, Osborne K, Wardle J. Enthusiasm for cancer screening in Great Britain: a general population survey.[Erratum appears in Br J Cancer. 2016 Jun 14;114(12):e14; PMID: 27228290]. *Br J Cancer* 2015;**112**:562–6.

21 Harte E, Maclure C, Martin A, *et al.* Reasons why people do not attend NHS Health Checks: A systematic review and qualitative synthesis. *Br J Gen Pract* 2017;:1–8. doi:https://doi.org/10.3399/bjgp17X693929

22 Palmer CK, Thomas MC, von Wagner C, *et al.* Reasons for non-uptake and subsequent participation in the NHS Bowel Cancer Screening Programme: a qualitative study. *Br J Cancer* 2014;**110**:1705–11. doi:10.1038/bjc.2014.125

23 Johnson TP, O??Rourke DP, Burris JE, *et al.* An Investigation of the Effects of Social

For peer review only - http://bmjopen.bmj.com/site/about/guidelines.xhtml

Desirability on the Validity of Self-Reports of Cancer Screening Behaviors. *Med Care* 2005;**43**:565–73. doi:10.1097/01.mlr.0000163648.26493.70

24 Allen JD, Bluethmann SM, Sheets M, *et al.* Women's responses to changes in U.S. preventive task force's mammography screening guidelines: Results of focus groups with ethnically diverse women. *BMC Public Health* 2013;**13**. doi:10.1186/1471-2458-13-1169

25 Rosenbaum L. Invisible Risks, Emotional Choices — Mammography and Medical Decision Making. *N Engl J Med* 2014;**371**:175–7.

26 Waller J, Bartoszek M, Marlow L, *et al.* Barriers to cervical cancer screening attendance in England: a population-based survey. *J Med Screen* 2009;**16**:199–204. doi:10.1258/jms.2009.009073

27 Health and Social Care Information Centre. Cervical Screening Programme, England – 2015-2016. https://digital.nhs.uk/data-and-information/publications/statistical/cervical-screening-annual/cervical-screening-programme-england-2015-2016 (accessed 10 Jul 2020).

28 Health and Social Care Information Centre. Breast Screening Programme, England – 2016-17. https://digital.nhs.uk/data-and-information/publications/statistical/breast-screening-programme/breast-screening-programme-england---2016-17#:~:text=Women between the ages of,better chance of successful treatment. (accessed 10 Jul 2020).

29 Logan RFA, Patnick J, Nickerson C, *et al.* Outcomes of the Bowel Cancer Screening Programme (BCSP) in England after the first 1 million tests. *Gut* 2012;**61**:1439–46.

For peer review only - http://bmjopen.bmj.com/site/about/guidelines.xhtml

doi:10.1136/gutjnl-2011-300843

30 Palan S, Schitter C. Prolific.ac—A subject pool for online experiments. *J Behav Exp Financ* 2018;**17**:22–7. doi:10.1016/j.jbef.2017.12.004

31 McCaffery K, Wardle J, Nadel M, *et al.* Socioeconomic variation in participation in colorectal cancer screening. *J Med Screen* 2002;**9**:104–8. doi:10.1136/jms.9.3.104

32 Forbes LJL, Simon AE, Warburton F, *et al.* Differences in cancer awareness and beliefs between Australia, Canada, Denmark, Norway, Sweden and the UK (the International Cancer Benchmarking Partnership): do they contribute to differences in cancer survival? *Br J Cancer* 2013;**108**:292–300. doi:10.1038/bjc.2012.542

33 Simon AE, Forbes LJL, Boniface D, *et al.* An international measure of awareness and beliefs about cancer: Development and testing of the ABC. *BMJ Open* 2012;**2**:1–8. doi:10.1136/bmjopen-2012-001758

FIGURE LEGENDS

Figure 1. Individual codes (facilitators and barriers) mapped onto overarching themes

SUPPLEMENTARY FILES

Supplementary File 1. Exploring attitudes towards kidney cancer screening survey (PDF 907kb)

For peer review only

Table 1. Participant characteristics

	UK (N=668)<br>n (%), mean (SD)
**Age**	
45-49	188 (28)
50-54	195 (29)
55-59	123 (18)
60-64	91 (14)
≥65	71 (11)
Mean (SD)	54.7 (7.0)
**Sex**	
Female	373 (54)
Male	295 (44)
**University education**	
Yes	288 (43)
No	380 (57)
**Ethnicity**	
White	653 (98)
Other	15 (2)
Missing	0
**General health measure**	
Excellent, very good, good	524 (78)
Fair, poor	144 (22)
**Smoking**	
Non-smoker	340 (51)
Ex-smoker	236 (35)
Current smoker	92 (14)
**BMI**	
Mean (SD)	27.4 (5.8)
Range	16.5-50.0
Missing	29 (4)
**Previous diagnosis of cancer?**	
Yes	34 (5)
No	634 (95)
**Family history of kidney cancer?**	
Yes	19 (3)
No	635 (95)
Missing	14 (2)
**Social group**	
ABC1	500 (75)
C2DE	136 (20)
Missing	32 (5)

For peer review only

Table 2. Illustrative quotes of themes

Qualitative theme	Barriers: Illustrative quotes	Facilitators: Illustrative quotes
**Personal health beliefs**	*"I have a healthy lifestyle and at a low risk for kidney cancer." (F, 55-59y, ultrasound)* *"There is no kidney cancer or cancer in my immediate family." (F, 45-49y, blood test)*	*"As my parent had kidney cancer, I would very much put myself forward to screening." (F, 60-64y, ultrasound)* *"Because life is precious, I want to live as long as possible." (M, 55-59y, CT)*
**Practicalities**	*"Because my mobility problems make it very difficult for me to travel anywhere." (F, 55-59y, ultrasound)* *"If it involved a specific visit, it's unlikely I would want to go." (M, 55-59y, urine test)*	*"It's no inconvenience at all to have any of the screening methods discussed" (M, 50-54y, urine test)* *"I have had ultrasound scans before, its quick and easy" (F, 45-49y, ultrasound scan)*
**Opinions of the test**	*"I would not wish to receive a dose of radiation to test for the extremely small chance of kidney cancer." (M, 65-69y, CT)* *"Really don't like needles, I'd opt for something else if it were available." (M, 45-49y, blood tests)*	*"A simple procedure and blood tests don't really alarm me at all" (M, 70-74y, blood test)* *"Easy to do when attending the doctors for something else and not painful in anyway" (F, 50-54y, urine test)*
**Attitudes towards screening**	*"Generally, I do not like medical appointments unless I need treatment." (M, 55-59y, blood tests)* *"There are a lot of cancers, you can't screen for all of them. If I had symptoms, I would see a GP." (F, 50-54y, ultrasound)*	*"Benefit of early detection outweighs minor inconvenience of screening" (M, 55-59y, blood test)* *"Would be foolish not to. If the disease has no symptoms and can be identified through screening, has the potential to save lives." (F, 55-59y, CT)*
**Cancer apprehension**	*"I would be too afraid of the results. Short of a transplant, what could be done anyway?" (F, 55-59y, urine test)* *"The stress of going to the hospital waiting for results is too great." (F, 60-64y, CT)*	*"Why waste a chance to 'set nerves at rest'?" (M, 55-59y, blood test)* *"High value of early detection or peace of mind" (M, 50-54y, blood test)*

For peer review only

For peer review only - http://bmjopen.bmj.com/site/about/guidelines.xhtml

Table 3. Facilitators and barriers of screening uptake across modalities, frequency of mention of codes and themes. The colour gradients shown indicate where each value falls within the range of data shown calculated within either the facilitator, or the barrier dataset. USS – ultrasound; HCP – healthcare professional; tx - treatment

Themes	Individual Codes	Blood *n*=589	Urine *n*=608	CT *n*=532	USS *n*=575	Total *n*=2304	Theme total
Personal Health beliefs	Life Extending / Improved survival	34	34	54	48	**204**	**224**
	Family history of cancer	2	1	2	3	**9**	
	Risk of kidney cancer	0	1	2	7	**11**	
Practicalities	Simple to complete	160	282	55	80	**859**	**1468**
	Little or no inconvenience	38	79	26	32	**254**	
	Test can be conducted at GP	22	32	0	4	**90**	
	Quick	42	57	32	43	**231**	
	Cost effective	8	10	4	2	**34**	
Opinions of the test	Painless	18	26	45	57	**172**	**803**
	Test non-invasive	6	23	27	53	**132**	
	Test is without risks	21	23	23	46	**136**	
	Comfortable having test	52	17	17	23	**126**	
	Have had test before	34	15	20	39	**123**	
	Effective test	25	24	14	13	**100**	
	Low radiation	0	1	10	2	**14**	
	Sufficient as a first test	8	9	1	2	**29**	
Attitudes towards screening	Early detection is a benefit	73	63	97	107	**403**	**928**
	Detection for better tx / more tx options	16	16	25	25	**98**	
	Sensible	42	44	54	73	**257**	
	For my general health / good	22	18	41	39	**138**	
	As part of other normal health checks	15	6	3	2	**32**	
	Want all screening / tests available	41	34	47	52	**208**	
	Benefits outweigh drawbacks of screening	84	13	72	28	**210**	
Cancer apprehension	Want to detect cancer	48	35	41	54	**213**	**485**
	Peace of mind /reassurance	46	43	66	74	**272**	
Other	General agreement	32	22	25	15	**116**	**129**
	Influence of HCP recommendation	3	1	4	4	13	
		Blood *n*=59	Urine *n*=29	CT *n*=111	USS *n*=56	Total *n*=255	Theme total
Personal health beliefs	No family history of cancer	1	1	1	1	**5**	**50**
	Perceived good health	2	2	0	2	**8**	
	No concern for kidney cancer	2	3	3	6	**17**	
	Believe themselves to be low risk	2	1	2	6	**12**	
	Other health concerns more important	1	1	2	3	**8**	
Practicalities	Inconvenience	3	3	7	4	**20**	**43**
	Do not want to travel to hospital	2	1	10	9	**23**	
Opinions of the test	Dislike test method (general)	21	1	9	1	**33**	**124**
	Painful	3	0	0	1	**4**	
	Invasive test	3	0	0	0	**3**	
	Risks of the test	1	0	53	1	**55**	
	Would have other testing first	2	0	11	2	**15**	
	Concern regarding inaccurate results	2	2	3	5	**14**	
Attitudes towards screening	Medical care for diagnosis / treatment	4	4	7	6	**25**	**70**
	Potential to lead to further tests	1	3	1	0	**8**	
	Not a good use of NHS resources	2	2	4	2	**12**	
	Do not want any screening	4	4	4	9	**25**	
Cancer Apprehension	Worry / fear about the results	6	6	6	7	**31**	**53**
	General fear	5	2	7	6	**22**	
Other	General negative comment	4	0	5	3	**12**	**36**
	Concerns regarding medical profession	2	1	3	3	**10**	
	Beliefs about treatment of kidney cancer	4	2	4	2	**14**	

For peer review only - http://bmjopen.bmj.com/site/about/guidelines.xhtml

Table 4. Subgroup analysis: participants likely to attend screening, frequency of mention of facilitators. The colour gradients shown indicate where each value falls within the range of data shown, calculated within each subgroup. USS – ultrasound; HCP – healthcare professional; tx - treatment

**Themes**	**Individual Codes**	**Participants likely to attend all screening modalities**						**Participants likely to attend screening by some, but not all screening modalities**					
		Blood	Urine	CT	USS	Total	Theme total	Blood	Urine	CT	USS	Total	Theme total
		n=510	*n*=499	*n*=510	*n*=495	*n*=2014		*n*=79	*n*=109	*n*=22	*n*=80	*n*=290	
Personal Health beliefs	Life Extending / Improved survival	30	33	54	46	163	180	4	1	0	2	7	8
	Family history of cancer	2	1	2	2	7		0	0	0	1	1	
	Risk of kidney cancer	0	1	2	7	10		0	0	0	0	0	
Practicalities	Simple to complete	131	216	51	61	459	793	29	66	4	19	118	215
	Little or no inconvenience	32	64	23	25	144		6	15	3	7	31	
	Test can be conducted at GP	13	22	0	2	37		9	10	0	2	21	
	Quick	33	40	30	31	134		9	17	2	12	40	
	Cost effective	6	7	4	2	19		2	3	0	0	5	
Opinions of the test	Painless	15	21	42	46	124	541	3	5	3	11	22	133
	Test non-invasive	6	16	24	35	81		0	7	3	18	28	
	Test is without risks	16	14	22	27	79		5	9	1	19	34	
	Comfortable having test	45	16	16	19	96		7	1	1	4	13	
	Have had test before	26	12	20	36	94		8	3	0	3	14	
	Effective test	17	14	14	10	55		8	10	0	3	21	
	Low radiation	0	1	10	1	12		0	0	0	1	1	
	Sufficient as a first test	6	7	1	1	15		2	2	0	1	5	
Attitudes towards screening	Early detection is a benefit	70	61	94	98	323	734	3	2	3	9	17	47
	Detection for better tx / more tx options	15	15	25	23	78		1	1	0	2	4	
	Sensible	40	41	51	68	200		2	3	3	5	13	
	For my general health / good	21	17	41	36	115		1	1	0	3	5	
	As part of other normal health checks	9	4	3	2	18		6	2	0	0	8	
	Want all screening / tests available	41	32	45	48	166		0	2	2	4	8	
	Benefits outweigh drawbacks of screening	74	13	68	25	180		10	0	4	3	17	
Cancer apprehension	Want to detect cancer	46	32	39	47	164	383	2	3	2	7	14	24
	Peace of mind / reassurance	45	40	66	68	219		1	3	0	6	10	
Other	General agreement	27	18	23	13	81	93	5	4	2	2	13	13
	Influence of HCP recommendation	3	1	4	4	12		0	0	0	0	0	

For peer review only - http://bmjopen.bmj.com/site/about/guidelines.xhtml

Table 5. Subgroup analysis: participants unlikely to attend screening, frequency of mention of barriers. The colour gradients shown indicate where each value falls within the range of data shown, calculated within each subgroup. USS – ultrasound.

Themes	Individual codes	Participants unlikely to attend any screening modalities						Participants unlikely to attend screening by some, but not all screening modalities					
		Blood	Urine	CT	USS	Total	Theme total	Blood	Urine	CT	USS	Total	Theme total
		n=27	*n*=27	*n*=27	*n*=28	*n*=109		*n*=32	*n*=2	*n*=84	*n*=28	*n*=146	
Personal health beliefs	No family history of cancer	1	1	1	1	**4**	**30**	0	0	0	0	**0**	**12**
	Perceived good health	2	2	0	2	**6**		0	0	0	0	**0**	
	No concern for kidney cancer	2	3	2	2	**9**		0	0	1	4	**5**	
	Believe themselves to be low risk	1	1	0	3	**5**		1	0	2	3	**6**	
	Other health concerns more important	1	1	1	3	**6**		0	0	1	0	**1**	
Practicalities	Inconvenience	0	2	2	0	**4**	**8**	3	1	5	4	**13**	**31**
	Do not want to travel to hospital	1	1	1	1	**4**		1	0	9	8	**18**	
Opinions of the test	Dislike test method (general)	2	1	1	0	**4**	**15**	19	0	8	1	**28**	**102**
	Painful	0	0	0	0	**0**		3	0	0	1	**4**	
	Invasive test	0	0	0	0	**0**		3	0	0	0	**3**	
	Risks of the test	1	0	4	0	**5**		0	0	45	1	**46**	
	Would have other testing first	0	0	0	0	**0**		2	0	11	2	**15**	
	Concern regarding inaccurate results	1	2	1	2	**6**		1	0	2	3	**6**	
Attitudes towards screening	Medical care for diagnosis / treatment	4	4	3	4	**15**	**37**	0	0	4	2	**6**	**20**
	Potential to lead to further tests	0	2	0	0	**2**		1	1	1	0	**3**	
	Not a good use of NHS resources	1	2	1	0	**4**		1	0	3	2	**6**	
	Do not want any screening	4	4	3	5	**16**		0	0	1	4	**5**	
Cancer Apprehension	Worry / fear about the results	3	6	4	5	**18**	**26**	3	0	2	2	**7**	**19**
	General fear	3	2	1	2	**8**		2	0	6	4	**12**	
Other	General negative comment	4	0	4	2	**10**	**28**	0	0	1	1	**2**	**5**
	Concerns regarding medical profession	1	1	2	3	1		1	0	1	0	**2**	
	Beliefs about treatment of kidney cancer	4	2	3	2	4		0	0	1	0	**1**	

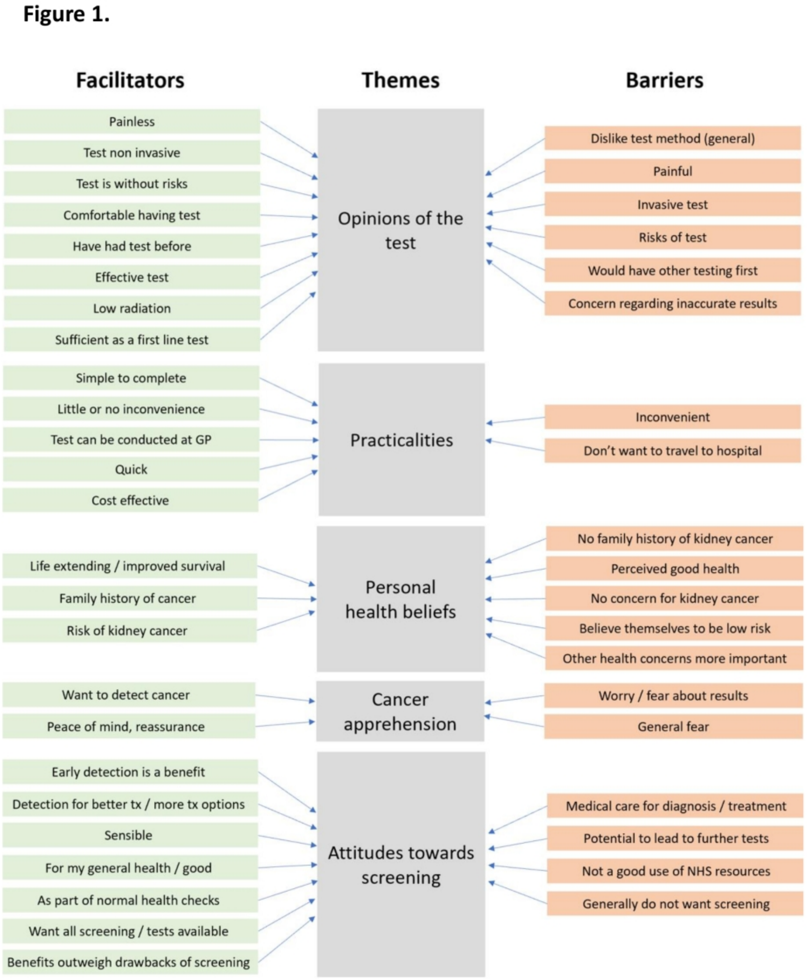

Figure 1. Individual codes (facilitators and barriers) mapped onto overarching themes

190x275mm (300 x 300 DPI)

Exploring attitudes towards kidney cancer screening survey

Question	Answer	Validation of question/reference
[Note that this content was delivered online so the formatting looked slightly different to in this document. The participants also did not see the headings in grey boxes or the references, but these are included to illustrate where the questions have come from.]		
Thank you for agreeing to complete this questionnaire. Please answer every question. If you are uncertain about how to answer a question, then please select the closest option.		
Demographic information *In this first section we would like to ask you a few questions about yourself. These questions allow us to make sure we are including people from a range of different backgrounds and see if different groups of people have different views. We will not be able to identify you from your answers.*		
How old are you?	• 45-49<br>• 50-54<br>• 55-59<br>• 60-64<br>• 65-69<br>• 70-74<br>• 75-79	
What is your ethnic group? Choose one option that best describes your ethnic group or background.	• White<br>• Mixed/Multiple ethnic group<br>• Asian/Asian British<br>• Black/African/Caribbean/Black British<br>• Other, please describe______	

For peer review only

What is your highest education level?	• Finished school at or before the age of fifteen<br>• Completed CSEs, O-levels or equivalent<br>• Completed A Levels or equivalent<br>• Completed further education but not a degree<br>• Completed a Bachelor's degree / Masters' degree / PHD<br>• Other (please specify) _______	Validated from Cancer Awareness Measure (CAM) toolkit, (pre-2014, as 2014 onwards has not been validated)[1]
Are you currently:	• Employed full-time<br>• Employed part-time<br>• Unemployed<br>• Self-employed<br>• Full-time homemaker<br>• Retired<br>• Still studying<br>• Disabled or too ill to work<br>• Prefer not to say	Validated from Cancer Awareness Measure (CAM) toolkit, (pre-2014, as 2014 onwards has not been validated)[1]
Please indicate to which occupational group the Chief Income Earner in your household belongs, or which group fits best. This could be you: the Chief Income Earner is the person in your household with the largest income. If the Chief Income Earner is retired and has a pension please answer for their most recent occupation. If the Chief Income Earner is not in paid employment but has been out of work for less than 6 months, please answer for their most recent occupation.	• Professional or higher technical work - work that requires at least degree-level qualifications (e.g. doctor, accountant, schoolteacher, university lecturer, social worker, systems analyst)<br>• Manager or Senior Administrator (e.g. company director, finance	Validated method of determining social class from National Readership Survey, used by Smits et al.,2018 to give ABC1

For peer review only

	manager, personnel manager, senior sales manager, senior local government officer)<br>• Clerical (e.g. clerk, secretary)<br>• Sales or Services (e.g. commercial traveller, shop assistant, nursery nurse, care assistant, paramedic)<br>• Foreman or Supervisor of Other Workers (e.g building site foreman, supervisor of cleaning workers)<br>• Skilled Manual Work (e.g. plumber, electrician, fitter, train driver, cook, hairdresser)<br>• Semi-Skilled or Unskilled Manual Work (e.g. machine operator, assembler, postman, waitress, cleaner, labourer, driver, bar-worker, call centre worker)<br>• Other (Please specify)<br>• Have never worked	(higher SES) and C2DE (lower SES) [2, 3]<br><br>CAM toolkit survey gives examples of occupations which I think fit into the ABC1C2DE framework.[1]
**Kidney cancer screening questions - Information given about kidney cancer potential screening tests**		
Kidney cancer screening tests<br><br>*The following section is about screening for kidney cancer. You will be shown information about kidney cancer and potential screening tests and then asked your views on different screening options.*<br><br>*Kidney cancer is the 7th most common cancer in the UK. There are around 12,600 new kidney cancer cases in the UK every year and 6000 deaths from kidney cancer. In about half of the cases of kidney cancer, there are no obvious symptoms at first. These cases are often picked up during tests*		

For peer review only

carried out for other reasons. Kidney cancer can often be cured, normally by surgery, if it is caught early. However, if it is not diagnosed until it has spread beyond the kidney, then a cure is much less likely. In the UK at the moment nearly a third of all cases of kidney cancer are diagnosed after the kidney cancer had spread beyond the kidneys.

Currently there is no screening programme for kidney cancer. There is potential for a screening programme to be developed in the future though. This may help detect kidney cancer at an early stage, making it easier to treat and reducing the number of people who die from kidney cancer. As with other screening programmes, it would involve having screening tests. If these tests find anything unusual, further tests (usually a detailed CT scan) are needed to confirm a possible kidney cancer. If they find a lump in the kidney then taking a small sample of that may be recommended to confirm or rule out cancer. Some patients wish to have surgery to remove the lump without confirming it is cancer and some of these lumps will not be cancer. As such some people may have an operation that is not required and others will be worried that they have cancer when they don't. There is also a chance that a cancer could be missed as no screening test is 100% reliable. Some people will therefore be told that they don't have cancer when they do.

Over the next few pages you will see some information about each of the potential screening tests. You will then be asked how much inconvenience, burden or worry you associate with each and how likely it is that you would attend for screening with each if you were invited. Please read through the information about the tests carefully so that you can use that information to help you answer those questions.

Ultrasound scan

- *Ultrasound scanning can be used to scan the kidneys. It is similar to the scan offered to pregnant women*
- *Ultrasound scans use high frequency sound waves to make a picture of organs inside your body.*
- *You would need to go to a clinic or hospital for the scan. At the scan you would be asked to lie down and show your abdomen (tummy) by lifting your top. You would not need to undress. A cool gel would be put on your skin.*
- *The ultrasound sensor would be moved over your skin on your lower back, in order to make a picture of each kidney which will appear on a screen.*
- *Ultrasound scans are not painful and there are no risks from the scans themselves. They do not involve exposure to radiation. Most ultrasound scans last between 15 and 45 minutes*
- *If all adults in the UK were offered ultrasound screening, around 3-4 out of every 1000 people screened would have an abnormal scan and require further investigations. Approximately half of those will have kidney cancer.*

For peer review only

Low-dose computed tomography (CT) scan

- *A low-dose computed tomography (CT) scan uses x-rays to make a detailed 3 dimensional (3D) picture of the kidneys. You would need to go to a clinic or hospital for the scan. At the scan you would lie on the machine couch on your back, and the couch slowly moves through the hole of the scanner. The scanner doesn't surround your whole body at once, so you shouldn't feel claustrophobic.*
- *This type of CT scan uses no dyes, no injections, and requires nothing to swallow by mouth*
- *The actual scan takes 1-2 minutes, and the appointment would normally take around 15 minutes.*
- *CT scans are quick, painless and generally safe.*
- *You would be exposed to X-ray radiation during the scan. Generally, the amount of radiation you're exposed to during each scan is the equivalent 6 months of exposure to natural radiation from the environment. It's thought exposure to radiation during CT scans could slightly increase your chances of developing cancer many years later, although this risk is thought to be very small (less than 1 in 2,000).*
- *If all people in the UK were offered screening with CT, around 3-4 out of every 1000 people screened would have an abnormal scan and require further investigations. Approximately half of those will have kidney cancer.*

Urine sample

- *In the future we hope to be able to test for biomarkers in the urine to identify people who are likely to have kidney cancer.*
- *This approach doesn't diagnose you with kidney cancer, but it is a simple way to find out if you need further tests.*
- *This would require you to provide a urine sample in a pot and either send it off or give to your GP. Your urine sample will then be tested to see if the biomarkers are present.*
- *There are no complications from the test itself but if a test is positive for the kidney cancer biomarkers you may have to have further tests.*
- *If all people in the UK were offered screening with a urine sample, estimates are that around 2-3 out of every 1000 people screened would have an abnormal test and require further investigations. Most of those will have kidney cancer.*

For peer review only

Blood test<br><br>• *In the future we hope to be able to test for biomarkers in the blood to identify people who are likely to have kidney cancer.*<br>• *This approach doesn't diagnose you with kidney cancer, but it is a simple way to find out if you need further tests.*<br>• *This would require you to have a blood test at your GP's surgery. The blood sample will tested at a laboratory to see if the biomarkers are present.*<br>• *A blood test can be slightly painful and leave a bruise.*<br>• *If the test is positive for the kidney cancer biomarkers you may have to have further tests.*<br>• *If all people in the UK were offered screening with a blood test, around 2-3 out of every 1000 people screened would have an abnormal test and require further investigations. Most of those will have kidney cancer.*		
If there was an **ultrasound based screening programme** and you were invited to have an **ultrasound scan** as part of that screening programme for kidney cancer, how likely do you think it is that you would choose to take part in the screening?	• Very likely<br>• Likely<br>• Unlikely<br>• Very unlikely	
Please describe in a few words why?	• Free text:_____	
If there was a **low-dose CT based screening programme** and you were invited to have a **low-dose CT scan** as part of a screening programme for kidney cancer, how likely do you think it is that you would choose to take part in the screening?	• Very likely<br>• Likely<br>• Unlikely<br>• Very unlikely	From Bowel cancer screening preferences survey (ongoing).
Please describe in a few words why?	• Free text:_____	
If there was a **urine sample based screening programme** and you were invited to give a **urine sample** as part of a screening programme for kidney cancer,	• Very likely<br>• Likely<br>• Unlikely	From Bowel cancer screening

For peer review only

how likely do you think it is that you would choose to take part in the screening?	• Very unlikely	preferences survey (ongoing).
Please describe in a few words why?	• Free text:_____	
If there was a **blood sample based screening programme** and you were invited to give a **blood sample** as part of a screening programme for kidney cancer, how likely do you think it is that you would choose to take part in the screening?	• Very likely<br>• Likely<br>• Unlikely<br>• Very unlikely	From Bowel cancer screening preferences survey (ongoing).
Please describe in a few words why?	• Free text:_____	
Thank you for completing the survey. Your time and responses are very much appreciated.		

References

1. Cancer Awareness Measure toolkit version 2.1 Cancer Research UK Cancer Awareness Measure (CAM) Toolkit (version 2.1). 2007. https://www.cancerresearchuk.org/sites/default/files/health_professional_cancer_awareness_measure_toolkit_version_2.1_09.02.11.pdf.

2. Smits SE, McCutchan GM, Hanson JA, Brain KE. Attitudes towards lung cancer screening in a population sample. Heal Expect. 2018; July:1–9.

3. Social Grade | National Readership Survey. http://www.nrs.co.uk/nrs-print/lifestyle-and-classification-data/social-grade/. Accessed 30 May 2019.

For peer review only

Consolidated criteria for reporting qualitative studies (COREQ): 32-item checklist

Developed from:
Tong A, Sainsbury P, Craig J. Consolidated criteria for reporting qualitative research (COREQ): a 32-item checklist for interviews and focus groups. *International Journal for Quality in Health Care*. 2007. Volume 19, Number 6: pp. 349 – 357

No. Item	Guide questions/description	Reported on Page #
**Domain 1: Research team and reflexivity**		
Personal Characteristics		
1. Inter viewer/facilitator	Which author/s conducted the inter view or focus group?	N/A
2. Credentials	What were the researcher's credentials? E.g. PhD, MD	8
3. Occupation	What was their occupation at the time of the study?	8
4. Gender	Was the researcher male or female?	8
5. Experience and training	What experience or training did the researcher have?	8
Relationship with participants		
6. Relationship established	Was a relationship established prior to study commencement?	N/A
7. Participant knowledge of the interviewer	What did the participants know about the researcher? e.g. personal goals, reasons for doing the research	N/A
8. Interviewer characteristics	What characteristics were reported about the inter viewer/facilitator? e.g. Bias, assumptions, reasons and interests in the research topic	N/A
**Domain 2: study design**		
Theoretical framework		
9. Methodological orientation and Theory	What methodological orientation was stated to underpin the study? e.g. grounded theory, discourse analysis, ethnography, phenomenology, content analysis	
Participant selection		
10. Sampling	How were participants selected? e.g. purposive, convenience, consecutive, snowball	7
11. Method of approach	How were participants approached? e.g. face-to-face, telephone, mail, email	7
12. Sample size	How many participants were in the study?	7
13. Non-participation	How many people refused to participate or dropped out? Reasons?	7
Setting		

14. Setting of data collection	Where was the data collected? e.g. home, clinic, workplace	5
15. Presence of non-participants	Was anyone else present besides the participants and researchers?	N/A
16. Description of sample	What are the important characteristics of the sample? e.g. demographic data, date	Table 1
Data collection		
17. Interview guide	Were questions, prompts, guides provided by the authors? Was it pilot tested?	7 and Supplementary file 1
18. Repeat interviews	Were repeat inter views carried out? If yes, how many?	N/A
19. Audio/visual recording	Did the research use audio or visual recording to collect the data?	N/A
20. Field notes	Were field notes made during and/or after the inter view or focus group?	N/A
21. Duration	What was the duration of the inter views or focus group?	N/A
22. Data saturation	Was data saturation discussed?	N/A
23. Transcripts returned	Were transcripts returned to participants for comment and/or correction?	N/A
**Domain 3: analysis and findings**		
Data analysis		
24. Number of data coders	How many data coders coded the data?	8
25. Description of the coding tree	Did authors provide a description of the coding tree?	Figure 1
26. Derivation of themes	Were themes identified in advance or derived from the data?	8
27. Software	What software, if applicable, was used to manage the data?	9
28. Participant checking	Did participants provide feedback on the findings?	N/A
Reporting		
29. Quotations presented	Were participant quotations presented to illustrate the themes/findings? Was each quotation identified? e.g. participant number	Table 2
30. Data and findings consistent	Was there consistency between the data presented and the findings?	Results
31. Clarity of major themes	Were major themes clearly presented in the findings?	11-14
32. Clarity of minor themes	Is there a description of diverse cases or discussion of minor themes?	11-14

For peer review only
